# Proximal tubular RAGE mediated the renal fibrosis in UUO model mice via upregulation of autophagy

**DOI:** 10.1038/s41419-022-04856-z

**Published:** 2022-04-23

**Authors:** Bohao Liu, Tianshi Sun, Huiling Li, Shuangfa Qiu, Yijian Li, Dongshan Zhang

**Affiliations:** 1 Department of Emergency Medicine, Second Xiangya Hospital, Changsha, China; 2 Emergency Medicine and Difficult Diseases Institute, Changsha, China; 3 Department of Spine Surgery, The Third Xiangya Hospital of Central South University, Changsha, Hunan China; 4 Department of Ophthalmology, Second Xiangya Hospital, Changsha, China; 5 Department of Urology, Second Xiangya Hospital, Changsha, China; 6 Department of Nephrology, Second Xiangya Hospital, Central South University, Changsha, Hunan China

**Keywords:** Autophagy, Urogenital diseases

## Abstract

Previous studies reported that RAGE participated in the process of kidney fibrosis, but the function and regulation pathway of RAGE in proximal tubular cells in this process remains unclear. Here, we found that expression of RAGE was increased by TGF-β1 treatment and unilateral ureteral obstruction (UUO). Knock down of RAGE ameliorated renal fibrosis by TGF-β1 treatment, the expression of vimentin, Collagen I&III, and fibronectin are decreased. Mechanistically, RAGE mediated TGF-β1-induced phosphorylation of Stat3 and directly upregulated the Atg7 to increase the level of autophagy, and ultimately resulting in renal fibrosis. Furthermore, PT-RAGE-KO mice reduced kidney fibrosis in UUO model via inhibiting Stat3/Atg7 axis by knocking down RAGE. Furthermore, the above findings were confirmed in kidney of patients with obstructive nephropathy. Collectively, RAGE in proximal tubular cells promotes the autophagy to increase renal fibrosis via upregulation of Stat3/Atg7 axis.

## Introduction

Chronic kidney disease (CKD) has a high prevalence rate [1, 2], which seriously affects human health. It is estimated that CKD existed in about more than 10% of adults in developed countries [1]. Kidney fibrosis is a major pathological feature of CKD. The existing treatments for renal fibrosis are only slightly effective or ineffective [3]. Emerging data suggest that the tubular epithelium regulates renal fibrosis [2, 4]. But mechanisms of tubular epithelium in renal fibrosis are still poorly understood.

Receptor for advanced glycation end products (RAGE) regulates the innate immune response via binding of numerous exogenous and endogenous ligands [5]. Recent studies report that it not only plays a pivotal role in early tissue repair in disease [6], but also is increased in the procession of occurrence and development of a variety diseases including cancer, diabetes, neurodegeneration [7]. Advanced glycation end products (AGEs) directly bind to RAGE, and leads to the increase of expression level of cytokines and growth factors, including vascular endothelial growth factor and connective tissue growth factor, finally results in glomerular injury [8–13]. Besides, previous study reported that global RAGE knockout mice reduced renal interstitial fibrosis via downregulation of transforming growth factor (TGF)-β [14]. But the function and mechanism of proximal tubular RAGE in renal fibrosis remain unclear according to available data.

Autophagy is a process which cytoplasmic components are degraded by lysosomes [15], The role of autophagy is associated with the type of cell or tissue and the experimental model [16]. For example, UUO-induced kidney fibrosis was exacerbated in PT-ATG5-KO autophagy deficiency mice or LC3(−/−) mice (deletion of LC3B) [17, 18], this was alleviated in PT-ATG7-KO autophagy deficiency mice in contrary [17]. The data indicated that the roles of autophagy in UUO-kidney fibrosis remain controversial. In addition, AGEs bind to RAGE and then induce autophagy in various diseases including heart disease and colorectal cancer [19–22], which suggested that RAGE partly mediates autophagy production. But the function and pathway of proximal tubular RAGE in UUO-induced autophagy are still unclear. According to the above literature, we hypothesize that proximal tubular RAGE can induce kidney fibrosis by regulating autophagy.

In our present study, we found that RAGE was induced by TGF-β1 and UUO, and then mediated autophagy to increase the renal fibrosis via STAT3/Atg7 axis in vitro and vivo. These results contribute to our understanding of the renoprotection by proximal tubular RAGE deletion in response to UUO.

## Materials and methods

### Antibodies and reagents

Anti-β-Tubulin (ab175186), anti-RAGE (ab216329), anti-fibronectin (ab2413), anti-Collagen І (ab138492), anti-Collagen III (ab184993), anti-vimentin (ab92547), and anti-α-SMA (ab124964) antibodies were purchased from Abcam (Cambridge, UK). Anti-Stat3 (9139), Phospho-Stat3 (4074), Atg7 (2631), and LC3-I/II (4108) were supplied by Cell Signaling Technology (MA, USA), while anti-p62, SQSTM1 (Cat No.18420-1-AP) were obtained from Proteintech (IL, USA). Recombinant human TGF-β1 (7754-BH) was obtained from R&D Systems (MN, USA). The RAGE siRNA and Atg7 siRNA were supplied by Santa Cruz Biotechnology. Atg7 and GFP- LC3-I/II plasmid was constructed by the Ruqi company (Guangzhou, China) [23].

### Animals

The proximal tubule-specific RAGE or Atg7-deletion mice were produced by crossing RAGE or Atg7 (flox/flox) mice (obtained from Dr Wang Lab and Dr Xiong Lab, respectively) with PEPCK-Cre mice [17]. C57BL/6 mice were obtained from Shanghai Model Organisms Center. The UUO model was constructed by ligating the left ureter in mice (male, 8–10 weeks of age) [2]. After obtaining ethical approval, the animals were experimented in compliance with the guidelines approved by the Animal Care Ethics Committee of Second Xiangya Hospital, China. The mice were housed at stable room temperature in a 12/12 h light/dark cycle and accessed to standard rodent chow and water. We estimate the sample size to 12 (*n* = 3 per group). The mice were divided into groups randomly without blinding by Random function of Excel (version 2019, Microsoft Corporation).

### Human samples

The project was approved by the Review Board of Second Xiangya Hospital, China, kidney biopsy samples were collected from obstructive nephropathy (Ob) patients as Ob group (*n* = 8) and paracancerous tissues of kidney as control group (*n* = 8). We announce that all experiments were performed in compliance with the Declaration of Helsinki principles, and also complied with the guidance of the Ministry of Science and Technology for the Review and Approval of Human Genetic Resources. We obtained the patient’s informed consent. The inclusion criteria of Ob were referred to a previous study [2].

### Cell culture, transfection, and treatments

The BUMPT cells were initially obtained from Drs. John Shwartz & William Lieberthal at Boston University [24]. BUMPT cells were grown in DMEM medium (Thermo-Fisher-Scientific) containing 10% FBS and antibiotics in a 37 °C incubator with 5% CO_2_. BUMPT cells were transfected with plasmids or siRNA using lipofection 2000 for twenty-four hours, followed by overnight starvation in a serum-free medium. Subsequently, the cells were treated with/without 5 ng/ml TGF-β1 for another 24 h. Bovine serum albumin (0.1%) was used as control [25].

### Histological, immunohistochemical, immunofluorescence, and Western blot analyses

Renal tissues were collected and stained with HE and Masson’s trichrome as previous described [2]. Immunohistochemical analysis was carried out using anti-RAGE (1:50), α-SMA (1:100), Collagen I/III (1:100), and fibronectin (1:100) in accordance with the previous instruction [2]. The immunofluorescence of puncta was performed following the standard process. Whole BUMPT cell or kidney tissue protein lysates were separated through SDS-PAGE, and then transferred to membrane and subsequently exposed to primary antibodies of RAGE, STAT3, p-STAT3, α-SMA, Collagen I/III, fibronectin, vimentin, and Atg7 following by the second antibody according to the standard procedure [26, 27].

### Establishment of PT-RAGE-KO mice

To explore the role of proximal tubular RAGE in renal fibrosis, we constructed a proximal tubules of RAGE-KO mouse model. The breeding instruction is denoted in Fig. S4A, floxed RAGE alleles in male mice (RAGE^f/f^ XY) were crossed with female phosphoenolpyruvate carboxy kinase-cAMP-response element (PEPCK-Cre) transgenic mice (RAGE^+/+^X^cre^X^cre)^. After the first-generation born, heterozygous female offsprings (RAGE^f/+^X^cre^X) were crossed with RAGE^f/+^X^cre^Y male mice to generate the proximal tubule RAGE wild-type (PT-RAGE-WT) and PT-RAGE-KO (RAGE^f/f^X^cre^Y) littermate mice. To identify genotypes, each mouse was subjected to three sets of PCR. PT-RAGE-KO mice has three genotypic features: (i) the 224 bp DNA fragment floxed allele is amplified; (ii) the 304 bp DNA fragment WT allele is insufficiently amplified; (iii) the 370 bp DNA fragment in Cre gene is amplified (Fig. S4B). Western blot analysis revealed that the RAGE expression level in the renal cortices of PT-RAGE-KO mice was decreased compared to PT-RAGE-WT under sham and UUO injury treatment (see Fig. S4C, D), this was further confirmed by the immunohistochemistry result (see Fig. S4E). The data indicated that tubular RAGE was knockdown in this conditional KO model.

### ChIP analysis

Chromatin immunoprecipitation (ChIP) assay was performed using commercial kit (Millipore, MA, USA) with primary antibodies against STAT3 [28]. The following primer pairs were employed to analyze the precipitated DNA through RT-qPCR: SBS1: 5′-CTGGCGGGGTTCA CTTAGGA-3′ and 5′-TCACAGTCCGGGGACACAAG-3′; SBS2: 5′-CCGAATCAACCTGCG TCTGC-3′, and 5′-CGGCCTCTGCTTCACTGAGT-3′; SBS3: 5′-TGTTGTTGTAGTGCGC ATGC-3′, and 5′-GGGGACCGAGTATAGACAGGT-3′; SBS4:5′-GGCCACGGAGTAAGCTTGT G-3′, and 5′-TGTGTCCGATTGCCTAGGCT-3′.

### Statistics

Statistical analyses were analyzed by GraphPad Prism 8.0. Quantitative data were described as mean ± standard deviation. Student’s t test was employed for comparing two groups, whereas one-way ANOVA was employed for comparing multiple groups. Level of statistical significance was set at *P* < 0.05.

## Results

### Expression of RAGE is upregulated by TGF-β1 in BUMPT cells, UUO in mice, and in the renal cortices of Ob-patients

**Fig. 1:**
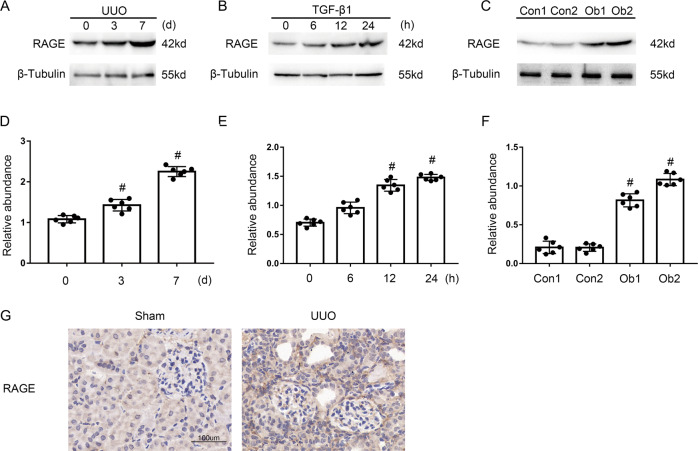
Induction of RAGE in BUMPT cells treated by TGF-β1 and the kidneys of UUO mice and Ob patients. **A**–**C** Immunoblot analysis of RAGE and β-Tubulin in BUMPT cells treated with or without TGF-β1 and the kidneys of UUO mice and Ob patients. **D**–**F** Analysis of the grayscale image between them. **G** Immunohistochemical staining of RAGE in the kidney of the Sham or UUO mice. Original magnification×400. Scale bar: 100 µM. Data are expressed as means ± s.d. (*n* = 6). #*P* < 0.05 versus 0 d & 0 h and Con group. Each experiment **A**–**C** was repeated six times independently with similar results. **B**–**F** indicate the statistical Student’s t test used (means ± s.d., *n* = 6, *P* < 0.05).

At the outset, the expression of RAGE in obstructive nephropathy (Ob) patients was upregulated significantly compared to the control group (Fig. 1C, F). Next, this founding was also confirmed in BUMPTs treated with/without TGF-β1 (Fig. 1A, D), which was the same as the results observed in the kidney tissues of UUO or sham mice (Fig. 1B, E). Moreover, the immunohistochemistry staining of RAGE verified our above results (Fig. 1G). RAGE expression was induced by TGF-β1 in BUMPTs, UUO in mice, and in the renal cortices of Ob-patients.

### RAGE mediates the TGF-β1-stimulated expression of fibronectin, vimentin, and Collagen I&III in BUMPTs

**Fig. 2:**
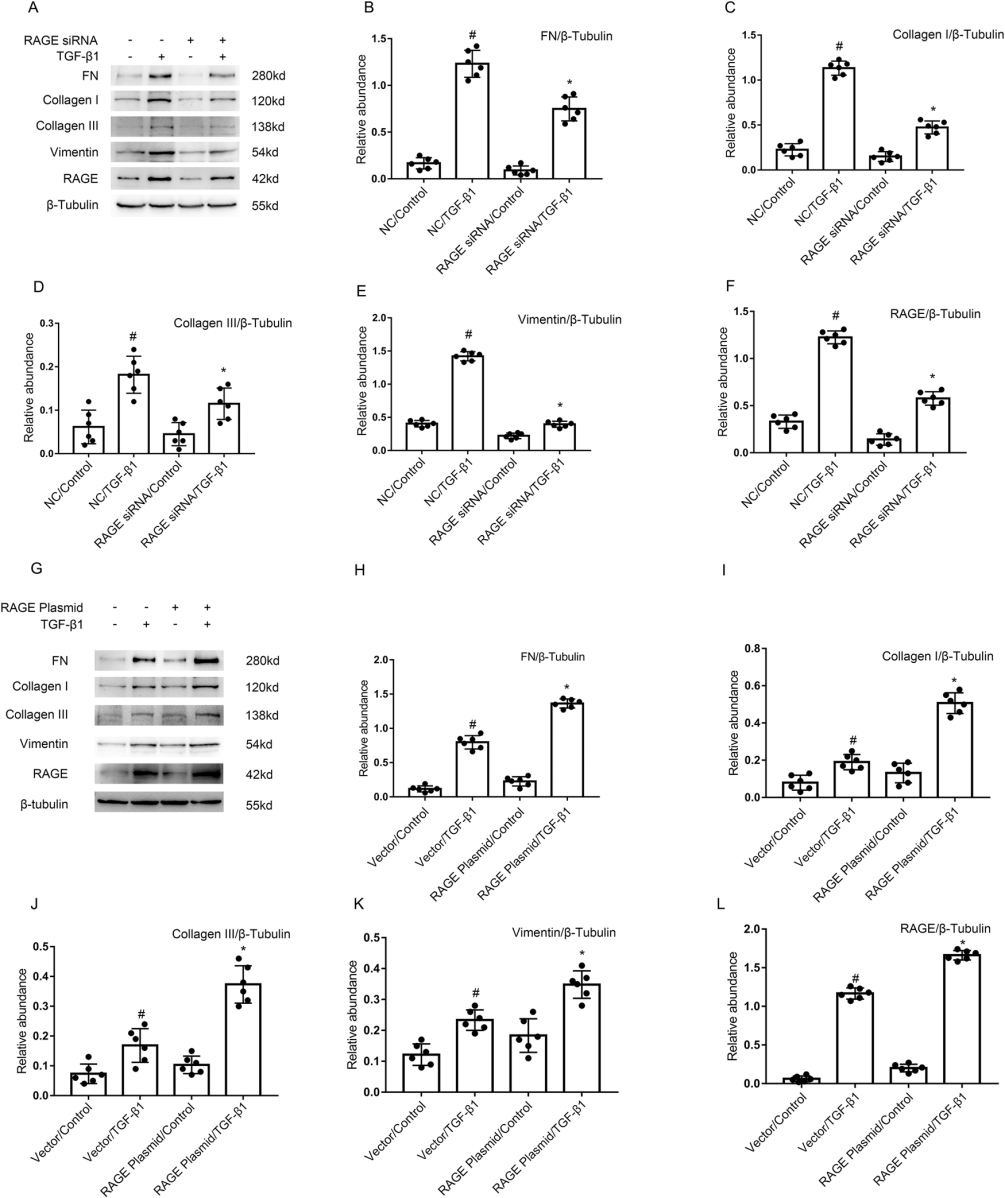
RAGE mediated the TGF-β1-induced expression of fibronectin & Col I&III & vimentin and RAGE in BUMPT cells. The plasmid and siRNA of RAGE were transfected into BUMPT cells and treated with or without 5 ng/ml TGF-β1 for 24 h. **A**, **G** Immunoblot analysis of fibronectin & Col I&III, vimentin, and RAGE. **B**–**F**, **H**–**L** Analysis of the gray scale image between them. Data are expressed as means ± s.d. (*n* = 6). #*P* < 0.05 versus NC or Vector with the control group. *P < 0.05 versus NC or Vector with the TGF-β1 group. Each experiment (**A**, **G**) was repeated six times independently with similar results. **B**–**F**, **H**–**L** indicate the statistical Student’s t test used (means ± s.d., *n* = 6, *P* < 0.05).

To determine the role of RAGE in renal fibrosis, BUMPTs were first transfected with RAGE siRNA or RAGE plasmid, and then administered with/without TGF-β1. The immunoblot results showed that TGF-β1-stimulated the enhancement of expression levels of fibronectin (FN), Collagen I&III, vimentin, and RAGE was attenuated by RAGE siRNA (see Fig. 2A–F), by the contrast, these changes were improved by the overexpression of RAGE plasmids (see Fig. 2G–L). The data demonstrated that RAGE at least partly mediated TGF-β1-stimulated the expression of fibronectin, vimentin, and Collagen I&III.

### RAGE mediated the TGF-β1-induced autophagy in BUMPTs

**Fig. 3:**
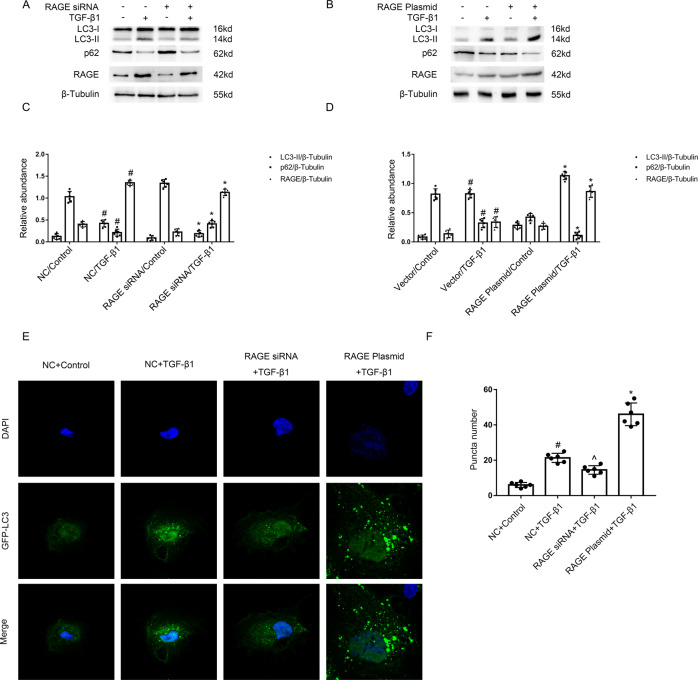
RAGE mediated the TGF-β1-induced autophagy in BUMPT cells. The plasmid and siRNA of RAGE were transfected into BUMPT cells or HK-2 cells and treated with or without 5 ng/ml TGF-β1 for 24 h. **A**, **B** Immunoblot analysis of LC3 & p62 & RAGE in BUMPT cells. **C**, **D** Analysis of the grayscale image between them. #*P* < 0.05 versus NC or Vector with the control group. *P < 0.05 versus NC or Vector with the TGF-β1 group. **E** After GFP-LC3 transfection, HK-2 cells were transfected with or without the plasmid or siRNA of RAGE, followed by the treatment with TGF-β1 for 24 h. The punctate accumulation of GFP-LC3 was visualized under fluorescence microscopy. **F** And the average number of GFP-LC3 dots per cell was then calculated. #*P* < 0.05 versus NC with the control group. ^*P* < 0.05 versus NC with the TGF-β1 group. *P < 0.05 versus NC with the TGF-β1 group. Each experiment **A**, **B**, **E** was repeated six times independently with similar results. **C**, **D**, **F** indicate the statistical Student’s t test used (means ± s.d., *n* = 6, *P* < 0.05).

Previous research has demonstrated that RAGE-mediated AGEs induced autophagy, but the role of RAGE in TGF-β1-triggered autophagy remains unclear. The immunoblot analysis demonstrated that TGF-β1-stimulated the upregulation of RAGE and LC3 II, and the reduction of p62 was markedly ameliorated by RAGE siRNA (see Fig. 3A, C), well, this was augmented by RAGE plasmids (see Fig. 3B, D). To observed autophagosome formation, GFP-LC3 was used to transfect HK-2 cells, which given a granular, punctate stain in the cytoplasm. As shown in Fig. 3E, F, the LC3 puncta numbers and intensity both improved after the treatment of TGF-β1, which was reduced by RAGE siRNA but augmented by RAGE plasmids. This finding was consistent with the immunoblot results. The data showed that RAGE mediated TGF-β1 induced autophagy.

### The RAGE mediated the TGF-β1-induced autophagy in BUMPT cells via STAT3/Atg7 axis

**Fig. 4:**
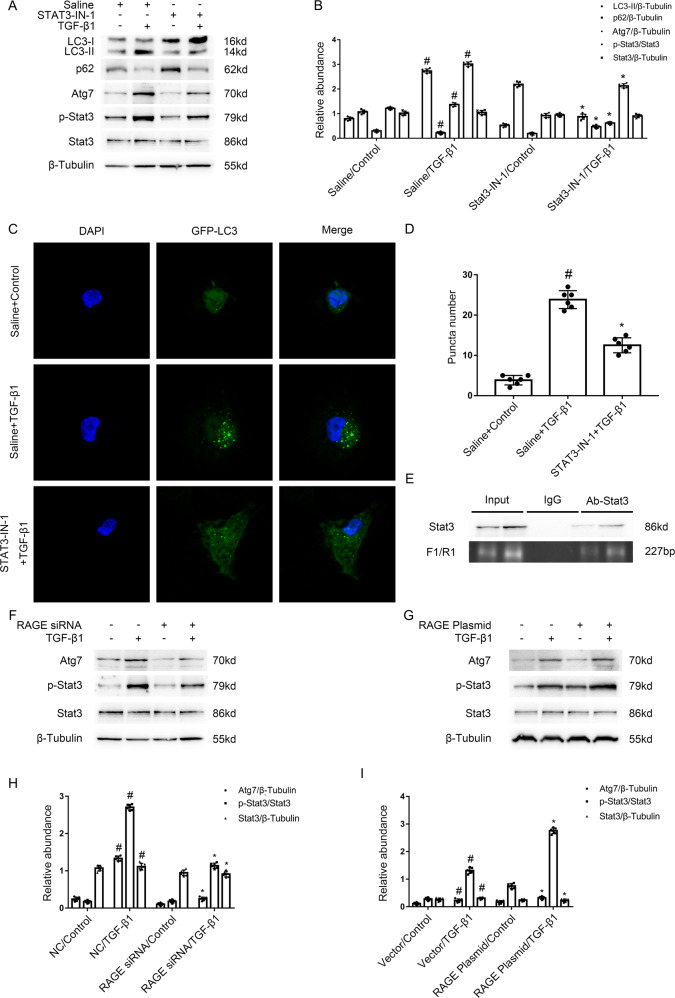
The RAGE mediated the TGF-β1-induced autophagy in BUMPT cells via STAT3/Atg7 axis. Immunoblotting results demonstrated that TGF-β1-induced the increasing of p-Stat3, Atg7, and LC3 II, and the reduction of p62 was notably attenuated by STAT3-IN-1, an STAT3 inhibitor. **A** Immunoblot analysis of LC3 & p62 & Atg7 & p-Stat3 & Stat3 in BUMPT cells. **B** Analysis of the grayscale image between them. #*P* < 0.05 versus Saline with the control group. *P < 0.05 versus Saline with the TGF-β1 group. **C** After GFP-LC3 transfection, HK-2 cells were transfected with or without STAT3-IN-1, followed by the treatment with TGF-β1 for 24 h. The punctate accumulation of GFP-LC3 was visualized under fluorescence microscopy. **D** The number of LC3 puncta per cell was calculated. #*P* < 0.05 versus Saline with the control group. *P < 0.05 versus Saline with the TGF-β1 group. **E** ChIP assays verified that a binding sites (a 227 bp fragment) of STAT3 existed in the promoter region of Atg7. The plasmid and siRNA of RAGE were transfected into BUMPT cells and treated with or without 5 ng/ml TGF-β1 for 24 h. **F**, **G** Immunoblot analysis of Atg7 & p-Stat3 & Stat3 in BUMPT cells. **H**, **I** Analysis of the grayscale image between them. #*P* < 0.05 versus NC or Vector with the control group. *P < 0.05 versus Saline with the TGF-β1 group. Each experiment **A**, **C**, **F**, **G** was repeated six times independently with similar results. **B**, **H**, **I** indicate the statistical Student’s t test used (means ± s.d., *n* = 6, *P* < 0.05).

The above finding revealed that RAGE promoted the renal fibrosis, however, the regulation mechanism remains largely unknown. In the current study, the immunoblotting results showed that TGF-β1-stimulated enhancement of p-Stat3, Atg7, and LC3 II, and the reduction of p62 was notably attenuated by STAT3-IN-1, an STAT3 inhibitor (see Fig. 4A, B). Furthermore, we observed that the administration of STAT3-IN-1 reduced the mean number of LC3 puncta in each cell vs TGF-β1 group (see Fig. 4C, D). Then, we investigated whether STAT3 is responsible for the upregulation of Atg7 during TGF-β1-induced autophagy in BUMPT cells. The predication result of JASPAR CORE database (http://jaspar.Genereg.net/) indicated that Atg7 promotor sequence contain four binding sites of STAT3. ChIP assays showed a binding site (a 227 bp fragment) for STAT3 in the promoter region of Atg7 (see Fig. 4E). Finally, the immunobloting results showed that TGF-β1-induced the Stat3/Atg7 signal pathway was attenuated by the RAGE siRNA (see Fig. 4F, H), oppositely, this was notably enhanced by the RAGE plasmid (see Fig. 4G, I). Collectively, the data revealed that RAGE/STAT3/Atg7 axis could mediate TGF-β1-triggered autophagy in BUMPT cells.

### Atg7 mediates TGF-β1-stimulated expression of fibronectin, vimentin, and Collagen I&III in BUMPT cells

**Fig. 5:**
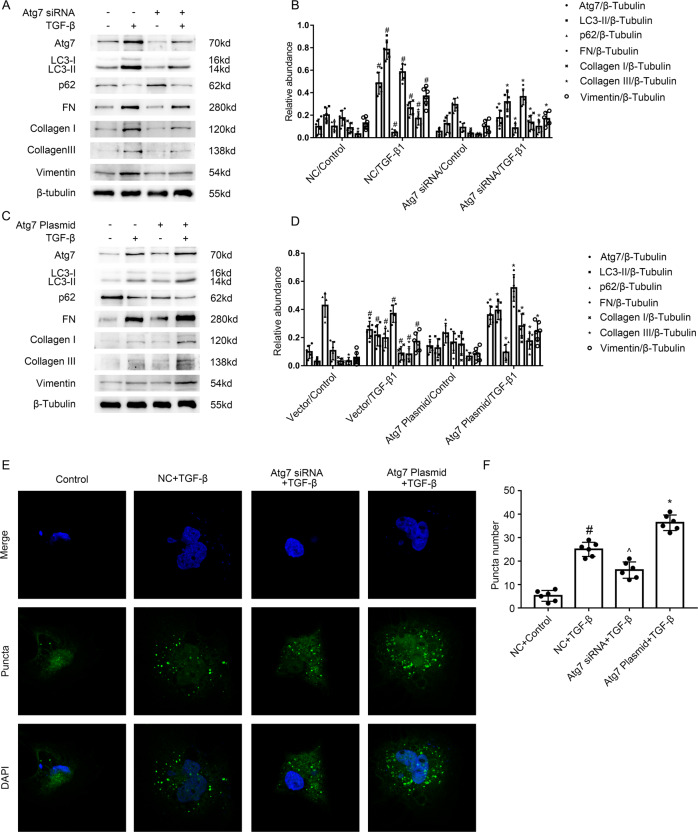
Atg7 mediated the TGF-β1-induced autophagy in BUMPT cells. The plasmid and siRNA of Atg7 were transfected into BUMPT cells and treated with or without 5 ng/ml TGF-β1 for 24 h. **A**, **C** Immunoblot analysis of Atg7 & LC3 & p62 & FN & Collagen I & Collagen III and Vimentin in BUMPT cells. **B**, **D** Analysis of the grayscale image between them. #*P* < 0.05 versus NC or Vector with the control group. *P < 0.05 versus NC or Vector with the TGF-β group. **E** After GFP-LC3 transfection, HK-2 cells were transfected with or without the plasmid or siRNA of ATG7, followed by the treatment with TGF-β1 for 24 h. The punctate accumulation of GFP-LC3 was visualized under fluorescence microscopy. **F** The number of LC3 puncta per cell was calculated. #*P* < 0.05 versus NC or Vector with the control group. ^*P* < 0.05 versus NC or Vector with the TGF-β group. *P < 0.05 versus NC or Vector with the TGF-β group. Each experiment **A**, **C**, **E** was repeated six times independently with similar results. **B**, **D**, **F** indicate the statistical Student’s t test used (means ± s.d., *n* = 6, *P* < 0.05).

The association between autophagy and renal fibrosis remains controversy [29]. UUO-induced kidney fibrosis was exacerbated in PT-ATG5-KO autophagy deficiency mice or LC3(−/−) mice(deletion of LC3B) [17, 18], in contrary, this was ameliorated in PT-ATG7-KO autophagy deficiency mice [17]. Here, we focused on the Atg7, a core autophagy-related protein. BUMPT cells transfected with Atg7 siRNA or Atg7 plasmid were treated with TGF-B1 as well as control vehicle. As displayed in Fig. 5A, C, TGF-β1-stimulated the increasing of Atg7, LC3 II, fibronectin, Collagen I&III, and vimentin as well as the reduction of p62 was markedly suppressed by the Atg7 siRNA (see Fig. 5A, B), by the contrast, this was reinforced by the Atg7 plasmid (see Fig. 5C, D). We further verified that TGF-β1-stimulated the enhancement of the mean number of LC3 puncta/cell was attenuated by the Atg7 siRNA, however, this was augmented by the Atg7 plasmid (see Fig. 5E, F). The data suggest that autophagy is at least partly responsible for the increasing of fibronectin, vimentin, and Collagen I&III induced by the TGF-β1.

### UUO-induced renal fibrosis was ameliorated in PT-ATG7-KO mice

**Fig. 6:**
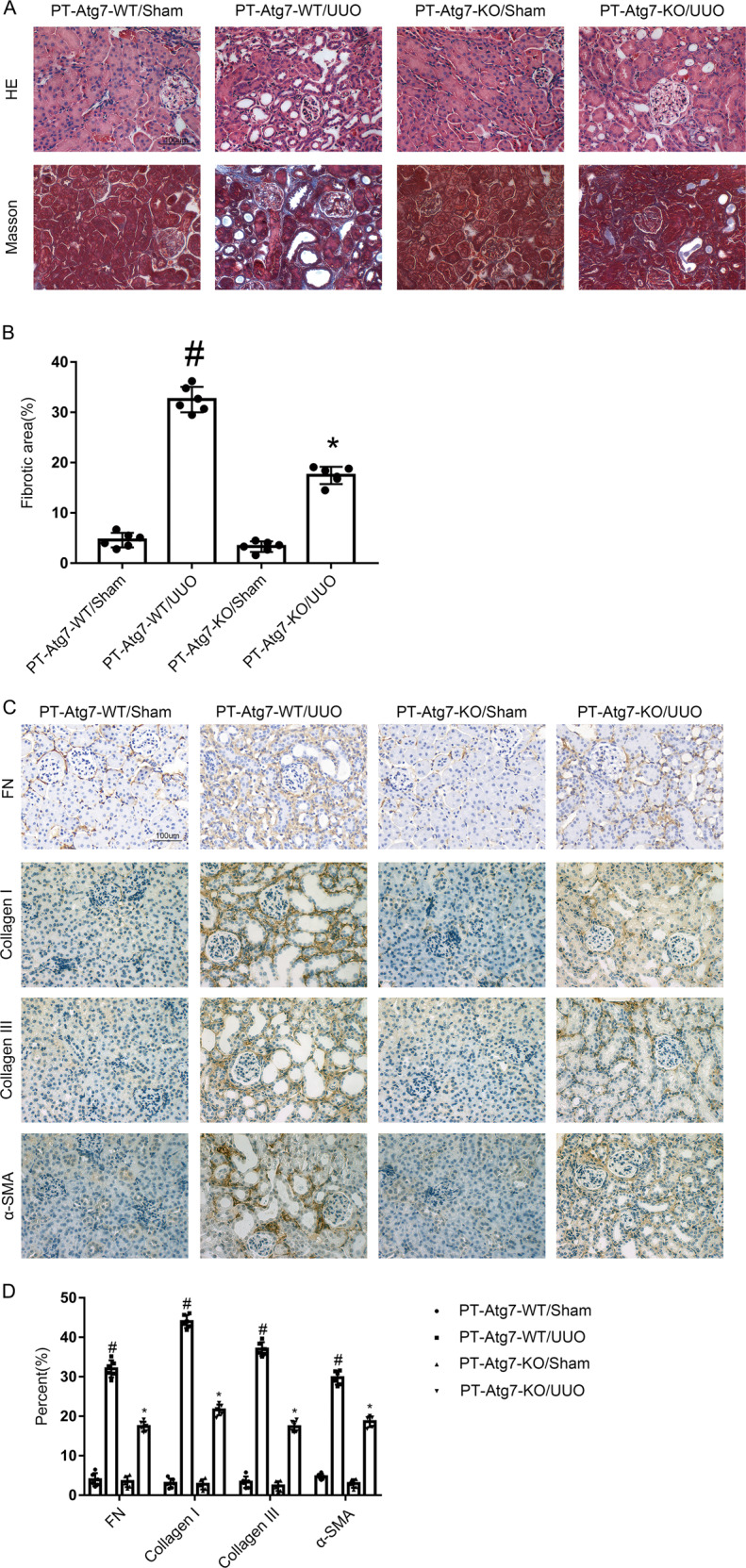
Amelioration of UUO-induced renal fibrosis in PT-ATG7-KO mice. The left ureter of PT-ATG7-KO and PT-ATG7-WT littermate mice was ligated for 7 days to establish a UUO model. **A** Hematoxylin and eosin staining and the representative Masson’s trichrome staining. **B** Quantification of tubulointerstitial fibrosis in the kidney cortex. **C** Immunohistochemistry of FN & Collagen I & Collagen III and α-SMA staining. **D** Quantification of the FN & Collagen I & Collagen III and α-SMA -positive cells in the kidney cortex. Original magnification ×400. Scale bar:100 µM. Data are expressed as means ± s.d. (*n* = 6). #*P* < 0.05 versus PT-ATG7-WT with the sham group. *P < 0.05 versus PT-ATG7-WT with the UUO group. Each experiment **A**, **C** was repeated six times independently with similar results. **B**, **D** indicate the statistical Student’s t test used (means ± s.d., *n* = 6, *P* < 0.05).

**Fig. 7:**
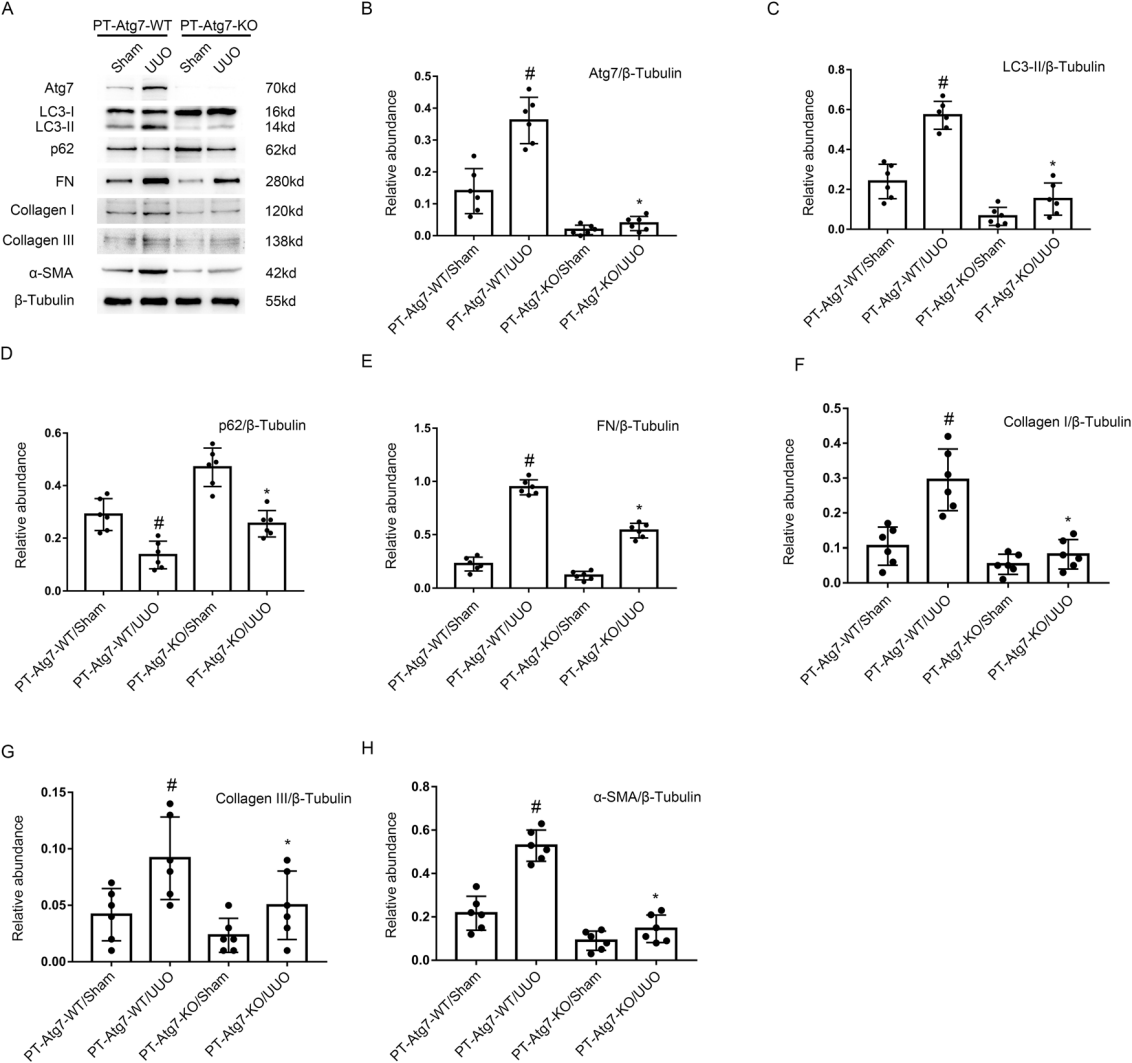
The UUO-induced expression of Col I&III and ɑ-SMA was attenuated due to the inhibition of autophagy in PT-ATG7-KO mice. The left ureter of PT-ATG7-KO and PT-ATG7-WT littermate mice was ligated for 7 days to establish a UUO model. Immunoblotting results demonstrated that the UUO induced the increasing of fibronectin & Col I&III, Vimentin, but the expression of them was reduced due to the downregulation of autophagy in PT-ATG7-KO mice. **A** Immunoblot analysis of Atg7, LC3, p62, and fibronectin & Col I&III, Vimentin. **B**–**H** Analysis of the grayscale image between them. Data are expressed as means ± s.d. (*n* = 6). #*P* < 0.05 versus PT-ATG7-WT with the sham group. *P < 0.05 versus PT-ATG7-WT with the UUO group. Each experiment (**A**) was repeated six times independently with similar results. **B**–**H** indicate the statistical Student’s t test used.

In order to confirm the role of Atg7 in renal fibrosis, PT-ATG7-KO mice was established, and then subjected to UUO model for 7 days. The results of HE and Masson staining demonstrated that UUO-induced tubular damage and renal interstitium fibrosis was ameliorated in PT-ATG7-KO mice (see Fig. 6A, B). Then, we performed immunochemical staining which showed that the expression of α-SMA, Collagen I&III, and fibronectin was upregulated in UUO model of PT-ATG7-WT mice, whereas it was notably ameliorated in PT-ATG7-KO mice (see Fig. 6C, D). The immunoblotting analysis revealed that UUO-induced the enhancement of Atg7, LC3 II, α-SMA, Collagen I&III, and fibronectin, and the reduction of p62 was markedly decreased in PT-ATG7-KO mice (see Fig. 7A–H). The data verified that Atg7 plays an essential role in UUO-induced renal fibrosis.

### UUO-induced kidney fibrosis is alleviated in PT-RAGE-KO mice via inhibition of STAT3/Atg7 axis

**Fig. 8:**
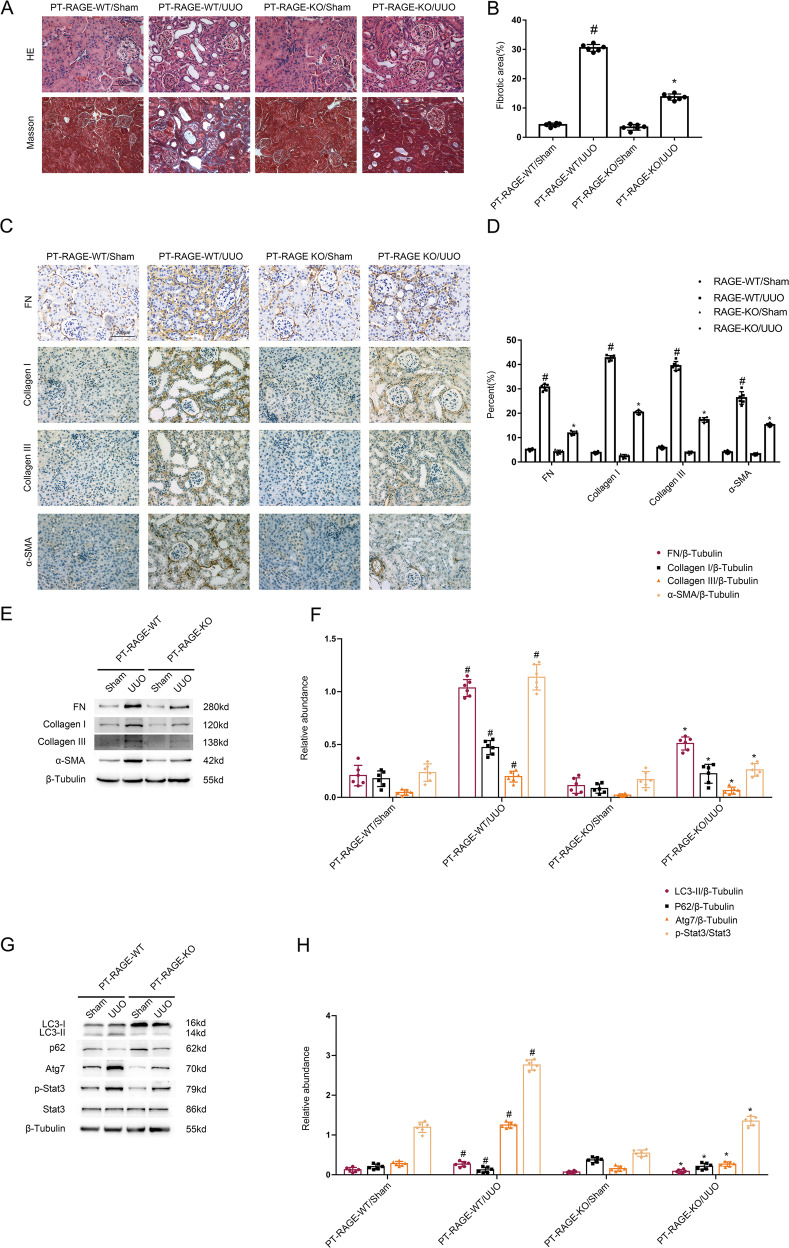
The UUO-induced renal fibrosis was ameliorated via the downregulation of Stat3 and autophagy in PT-RAGE-KO mice. The left ureter of PT-RAGE-KO and PT-RAGE-WT littermate mice was ligated for 7 days to establish a UUO model. **A** Hematoxylin and eosin staining and the representative Masson’s trichrome staining. **B** Quantification of tubulointerstitial fibrosis in the kidney cortex. **C** Immunohistochemistry of FN & Collagen I & Collagen III and α-SMA staining. **D** Quantification of the FN & Collagen I & Collagen III and α-SMA -positive cells in the kidney cortex. **E**, **G** Immunoblot analysis of fibronectin & Col I&III, α-SMA and p-Stat3 & Stat3, Atg7, LC3, p62. **F**, **H** Analysis of the grayscale image between them. Original magnification ×400. Scale bar:100 µM. Data are expressed as means ± s.d. (*n* = 6). #*P* < 0.05 versus PT-RAGE-WT with the sham group. *P < 0.05 versus PT-RAGE-WT with the UUO group. Each experiment **A**, **C** was repeated six times independently with similar results. **B**, **D** indicate the statistical Student’s t test used (means ± s.d., *n* = 6, *P* < 0.05). Data are expressed as means ± s.d. (*n* = 6). #*P* < 0.05 versus sham group. *P < 0.05 versus PT-RAGE-WT with the UUO group.

The PT-RAGE-KO and PT-RAGE-WT littermate mice were subjected to UUO model for 7 days. The results of HE and Masson staining demonstrated that UUO-induced tubular damage and renal interstitium fibrosis was alleviated in PT-RAGE-KO mice (see Fig. 8A, B). The data of immunochemical staining showed that UUO-induced the expression of α-SMA, Collagen I&III, and fibronectin was remarkably decreased in PT-RAGE-KO mice (Fig. 8C, D). The immunoblotting analysis demonstrated that UUO-induced induced the increasing of α-SMA, Collagen I&III, fibronectin, Atg7, LC3 II as well as the reduction of p62 was markedly reversed in PT-RAGE-KO mice (Fig. 8E–H). The data confirmed that Proximal Tubular RAGE mediated the renal fibrosis in UUO model mice via upregulation of autophagy.

### RAGE mediates TGF-β1-induced kidney fibrosis depended on the Atg7 in vitro *and* vivo

To further explore whether RAGE promotes the renal fibrosis via autophagy during TGF-β1 treatment, we carried out the following experiments. Firstly, RAGE siRNA significantly ameliorated the TGF-β1-stimulated the expression of α-SMA, Collagen I&III, and fibronectin in BUMPTs, which was markedly reversed by the overexpression of Atg7 (see Fig. S1A, B). Consistently, the results of HE and Masson staining demonstrated that PT-RAGE-KO attenuated UUO-induced tubular damage and renal interstitium fibrosis, which was attenuated by the injection of Atg7 plasmid (see Fig. S1C, D). The Western blot data showed that PT-RAGE-KO mice not only suppressed the UUO-induced kidney fibrosis but also the expression of α-SMA, Col I&III, and fibronectin, which confirmed the above pathological results (see Fig. S1E, F). Secondly, the data of HE and Masson staining revealed that UUO-induced tubular damage and renal interstitium fibrosis was alleviated in PT-Atg7-KO mice which was injected with RAGE Plasmid (see Fig. S2C, D). Atg7 siRNA remarkably decreased the TGF-β1-stimulated the expression of α-SMA, Collagen I&III, and fibronectin in BUMPTs, this was not reversed by the overexpression of RAGE (see Fig. S2A, B). Consistently, PT-Atg7-KO mice not only reduced the UUO-induced kidney fibrosis but also the expression of α-SMA, Col I&III, and fibronectin, this was also not reversed by the overexpression of RAGE (see Fig. S2E, F). The data demonstrated that RAGE induced the renal fibrosis depended on the autophagy in vitro and vivo models.

### RAGE/Stat3/Atg7 axis mediated renal fibrosis in patients with Ob

The data of HE and Masson staining showed that patients with Ob-triggered tubular damage and renal interstitium fibrosis than the paracancerous tissues of kidney (see Fig. S3A, B). we showed the patients’ clinical information in Table 1. Immunoblot data also revealed that patients with Ob-induced the expression of α-SMA, Collagen I&III, and fibronectin, Atg7, RAGE, LC3II, and p-stat3 as well as the decreasing of p62 than the paracancerous tissues of kidney (see Fig. S3C–F).

**Table 1:** The basic clinical information of renal carcinoma (RC) patients and Ob patients.

Characteristics	RC (*n* = 8)	Ob (*n* = 8)	*P* value
*Gender*			
Male	4	3	
Female	4	5	
Age(years)(mean)[s.d.]	54.00[3.03]	58.17[11.34]	0.2693
BMI(mean)[s.d.]	24.21[4.09]	23.34[2.88]	0.6788
*Degree of hydronephrosis*			
0	8(100%)	0	
1	0	0	
2	0	0	
3	0	2(25%)	
4	0	6(75%)	
Diagnosis	Renal carcinoma	Hydronephrosis	
*Operation side*			
Left	3	4	
Right	5	4	
Blood urea nitrogen (mM) (mean)[s.d.]	5.42[1.40]	5.74[1.39]	0.6962
Serum creatinine (mM) (mean)[s.d.]	74.98[12.03]	85.18[10.47]	0.1482

## Discussion

Previous studies demonstrated that global RAGE knock out attenuated that renal fibrosis [14, 30]. This study for the first time demonstrated that proximal tubular RAGE also attenuated the kidney fibrosis induced by TGF-β1 in vitro and UUO in vivo. Mechanistically, RAGE mediated the TGF-β1-triggered Stat3 activation and then promoted autophagy to increase renal fibrosis via upregulation of Atg7. The data suggested that tubular RAGE may be considered as a therapy target.

RAGE have multi-ligand pattern of AGEs, S100 proteins, high mobility group box1(HMGB1), lysophosphatidic acid (LPA), amyloid beta peptide (Aβ), islet amyloid polypeptide (IAPP), and macrophage 1-antigen (Mac-1) [31]. Several studies have shown that the AGEs-RAGE pathway plays vital roles in the progression of various kidney disorders including hypertensive nephropathy, diabetic nephropathy, lupus nephritis, obesity-related glomerulopathy, amyloidosis, ADPKD, and septic AKI [32–39]. The global knock out or inhibition of RAGE were used to block the AGEs-RAGE pathway in the above studies. The above references suggested that AGEs may be a key ligand of RAGE in mice UUO model. However, the role of proximal tubular RAGE remains unclarified. In the current study, we firstly assessed the increasing of RAGE in the kidneys of Ob patients and UUO mice likewise in TGF-β1-treated BUMPTs (see Fig. 1). Secondly, we demonstrated that tubular RAGE mediated the renal fibrosis, which was supported by the following evidences: (1) RAGE siRNA ameliorated TGF-β1-stimulated the expression of vimentin, collagen I&III, and fibronectin (see Fig. 2). (2) PT-RAGE-KO mice notably ameliorated UUO-induced renal fibrosis (see Fig. 8).

Previous research has reported that RAGE could regulate the formation of autophagy. For example, Dr Gao et al. reported that RAGE mediated the autophagy via upregulation of pp65-NFκB and BNIP3 [19] in pressure overload-induced heart failure. Dr Hou et al. found that RAGE mediated AGEs-induced autophagy in cardiomyocyte injury via PI3K/AKT/mTOR pathway [20]. Dr Huang et al. demonstrated that RAGE promoted the autophagy in colorectal cancer via ERK/Drp1 phosphorylation [21]. Dr. Meng et al. verified that RAGE promoted the autophagy in diabetes-associated osteoporosis through Raf/MEK/ERK signaling pathway [22]. In the present study, we found that RAGE mediated autophagy, which was demonstrated by the following findings: (1) TGF-β1-triggered autophagy was attenuated by the RAGE siRNA, which was improved by the overexpression of RAGE (see Fig. 3). (2) PT-RAGE-KO mice notably ameliorated the UUO-induced autophagy (see Fig. 8). Furthermore, we found that Stat3/Atg7 signal pathway is responsible for the autophagy production mediated by tubular RAGE during TGF-β1 and UUO treatment. The following evidences supported the above finding: (1) Inactivation of Stat3 signal pathway reduced TGF-β1 induced autophagy via downregulation of Atg7 (see Fig. 4A–D). (2) ChIP analysis for the first time indicated that Stat3 directly binds the promoter of Atg7 (see Fig. 4E). (3) The Stat3/Atg7 signal pathway was suppressed by the RAGE siRNA or PT-RAGE-KO (see Figs. 4F–I and 8E–H), by the contrast, this was improved by RAGE overexpression. In addition, this signal pathway was also upregulated expressed in OB patients (see Fig. S3). Together, these results support that RAGE/Stat3/Atg7 axis mediated TGF-β1 and UUO-induced autophagy.

The roles of autophagy in renal fibrosis still need to be clarified. The research from two groups reported that PT-ATG5-KO autophagy deficiency or global knock out of LC3B exacerbated UUO-induced renal fibrosis [17, 18]. However, Dr Dong et al. found that PT-ATG7-KO autophagy deficiency mice attenuated UUO-induced renal fibrosis [17]. In this study, we found that PT-ATG7-KO autophagy deficiency mice notably reduced the renal fibrosis in UUO model (see Figs. 6, 7). In addition, TGF-β1-stimulated the expression of vimentin, collagen I&III, and fibronectin was notably reduced by the Atg7 knockdown, by contrast, this was augmented by the overexpression of Atg7 (see Fig. 5). Our results are consistent with the Dr Dong’s finding. Finally, we found that overexpression of Atg7 diminished the protective role of RAGE siRNA or PT-RAGE-KO on kidney fibrosis induced by TGF-β1 or UUO (see Fig. S1), however, the protective role of Atg7 siRNA or PT-ATG7-KO on kidney fibrosis induced by TGF-β1 or UUO was not enhanced by the overexpression of RAGE. The data suggested that RAGE promoted autophagy to exacerbate renal fibrosis during TGF-β1 or UUO treatment.

In conclusion, we found that proximal tabular RAGE-mediated renal fibrosis in vitro and vivo. Mechanistically, TGF-β1 stimulated the RAGE and then activated STAT3 to increase autophagy via directly upregulation of Atg7, and then promoted the progression of renal fibrosis. Our study suggests that the RAGE/STAT3/Atg7 axis can serve as a therapeutic target of kidney fibrosis.

## Supplementary information

supplementary

Checklist

Similarity Report

Original Data File

## Data Availability

The datasets used and/or analyzed during the current study are available from the corresponding author on reasonable request.

## References

[1] López-Novoa JM, Martínez-Salgado C, Rodríguez-Peña AB, López-Hernández FJ. Common pathophysiological mechanisms of chronic kidney disease: Therapeutic perspectives. Pharm Ther. 2010;128:61–81. 10.1016/j.pharmthera.2010.05.006 20600306

[2] Li X, Pan J, Li H, Li G, Liu X, Liu B, et al. DsbA-L mediated renal tubulointerstitial fibrosis in UUO mice. Nat Commun. 2020;11:4467. 32948751 10.1038/s41467-020-18304-z PMC7501299

[3] Nogueira A, Pires MJ, Oliveira PA. Pathophysiological mechanisms of renal fibrosis: A review of animal models and therapeutic strategies. In Vivo. 2017;31:1–22. 28064215 10.21873/invivo.11019 PMC5354133

[4] Zeisberg M, Kalluri R. The role of epithelial-to-mesenchymal transition in renal fibrosis. J Mol Med. 2004;82:175–81. 14752606 10.1007/s00109-003-0517-9

[5] Mahajan N, Dhawan V. Receptor for advanced glycation end products (RAGE) in vascular and inflammatory diseases. Int J Cardiol. 2013;168:1788–94. 23722052 10.1016/j.ijcard.2013.05.013

[6] Rabbani N, Thornalley PJ. Advanced glycation end products in the pathogenesis of chronic kidney disease. Kidney Int. 2018;93:803–13. 29477239 10.1016/j.kint.2017.11.034

[7] Yamamoto M, Sugimoto T. Advanced glycation end products, diabetes, and bone strength. Curr Osteoporos Rep. 2016;14:320–6. 27704396 10.1007/s11914-016-0332-1 PMC5104767

[8] Hagiwara S, Sourris K, Ziemann M, Tieqiao W, Mohan M, McClelland AD, et al. RAGE deletion confers renoprotection by reducing responsiveness to transforming growth factor-β and increasing resistance to apoptosis. Diabetes. 2018;67:960–73. 29449307 10.2337/db17-0538

[9] Ranzinger J, Rustom A, Heide D, Morath C, Schemmer P, Nawroth PP, et al. The receptor for advanced glycation end-products (RAGE) plays a key role in the formation of nanotubes (NTs) between peritoneal mesothelial cells and in murine kidneys. Cell Tissue Res. 2014;357:667–79. 24870978 10.1007/s00441-014-1904-y

[10] Hong J, Li G, Zhang Q, Ritter J, Li W, Li PL. D-Ribose induces podocyte NLRP3 inflammasome activation and glomerular injury via AGEs/RAGE pathway. Front Cell Dev Biol. 2019;7:259. 31737627 10.3389/fcell.2019.00259 PMC6831643

[11] D’Agati V, Yan SF, Ramasamy R, Schmidt AM. RAGE, glomerulosclerosis, and proteinuria: Roles in podocytes and endothelial cells. Trends Endocrinol Metab. 2010;21:50–6. 19783154 10.1016/j.tem.2009.07.003

[12] Meek RL, LeBoeuf RC, Saha SA, Alpers CE, Hudkins KL, Cooney SK, et al. Glomerular cell death and inflammation with high-protein diet and diabetes. Nephrol Dial Transpl. 2013;28:1711–20. 10.1093/ndt/gfs579 PMC3707525 23314315

[13] Hou B, Qiang G, Zhao Y, Yang X, Chen X, Yan Y, et al. Salvianolic acid A protects against diabetic nephropathy through ameliorating glomerular endothelial dysfunction via inhibiting AGE-RAGE signaling. Cell Physiol Biochem. 2017;44:2378–94. 29262395 10.1159/000486154

[14] Gasparitsch M, Arndt AK, Pawlitschek F, Oberle S, Keller U, Kasper M, et al. RAGE-mediated interstitial fibrosis in neonatal obstructive nephropathy is independent of NF-κB activation. Kidney Int. 2013;84:911–9. 23677242 10.1038/ki.2013.171

[15] Tanida I, Ueno T, Kominami E. LC3 and autophagy. Methods Mol Biol. 2008;445:77–88. 18425443 10.1007/978-1-59745-157-4_4

[16] Tanida I, Ueno T, Kominami E. LC3 conjugation system in mammalian autophagy. Int J Biochem Cell Biol. 2004;36:2503–18. 15325588 10.1016/j.biocel.2004.05.009 PMC7129593

[17] Li H, Peng X, Wang Y, Cao S, Xiong L, Fan J, et al. Atg5-mediated autophagy deficiency in proximal tubules promotes cell cycle G2/M arrest and renal fibrosis. Autophagy 2016;12:1472–86. 27304991 10.1080/15548627.2016.1190071 PMC5082781

[18] Ding Y, Kim S, Lee SY, Koo JK, Wang Z, Choi ME. Autophagy regulates TGF-β expression and suppresses kidney fibrosis induced by unilateral ureteral obstruction. J Am Soc Nephrol. 2014;25:2835–46. 24854279 10.1681/ASN.2013101068 PMC4243349

[19] Gao W, Zhou Z, Liang B, Huang Y, Yang Z, Chen Y, et al. Inhibiting receptor of advanced glycation end products attenuates pressure overload-induced cardiac dysfunction by preventing excessive autophagy. Front Physiol. 2018;9:1333. 30319444 10.3389/fphys.2018.01333 PMC6165873

[20] Hou X, Hu Z, Xu H, Xu J, Zhang S, Zhong Y, et al. Advanced glycation endproducts trigger autophagy in cadiomyocyte via RAGE/PI3K/AKT/mTOR pathway. Cardiovasc Diabetol. 2014;13:78. 24725502 10.1186/1475-2840-13-78 PMC3998738

[21] Huang CY, Chiang SF, Chen WT, Ke TW, Chen TW, You YS, et al. HMGB1 promotes ERK-mediated mitochondrial Drp1 phosphorylation for chemoresistance through RAGE in colorectal cancer. Cell Death Dis. 2018;9:1004. 30258050 10.1038/s41419-018-1019-6 PMC6158296

[22] Meng HZ, Zhang WL, Liu F, Yang MW. Advanced glycation end products affect osteoblast proliferation and function by modulating autophagy via the receptor of advanced glycation end products/Raf protein/mitogen-activated protein kinase/extracellular signal-regulated kinase kinase/extracellular signal-regulated kinase (RAGE/Raf/MEK/ERK) pathway. J Biol Chem. 2015;290:28189–99. 26472922 10.1074/jbc.M115.669499 PMC4653677

[23] Xu L, Li X, Zhang F, Wu L, Dong Z, Zhang D. EGFR drives the progression of AKI to CKD through HIPK2 overexpression. Theranostics. 2019;9:2712–26. 31131063 10.7150/thno.31424 PMC6526000

[24] Bhatt K, Zhou L, Mi QS, Huang S, She JX, Dong Z. MicroRNA-34a is induced via p53 during cisplatin nephrotoxicity and contributes to cell survival. Mol Med. 2010;16:409–16. 20386864 10.2119/molmed.2010.00002 PMC2935954

[25] Li X, Pan J, Li H, Li G, Liu B, Tang X, et al. DsbA-L interacts with VDAC1 in mitochondrion-mediated tubular cell apoptosis and contributes to the progression of acute kidney disease. EBioMedicine. 2022;76:103859. 35124430 10.1016/j.ebiom.2022.103859 PMC8829058

[26] Cheng X, Ai K, Yi L, Liu W, Li Y, Wang Y, et al. The mmu_circRNA_37492/hsa_circ_0012138 function as potential ceRNA to attenuate obstructive renal fibrosis. Cell Death Dis. 2022;13:207. 35246505 10.1038/s41419-022-04612-3 PMC8897503

[27] Ai K, Pan J, Zhang P, Li H, He Z, Zhang H, et al. Methyl-CpG-binding domain protein 2 contributes to renal fibrosis through promoting polarized M1 macrophages. Cell Death Dis. 2022;13:125. 35136032 10.1038/s41419-022-04577-3 PMC8826408

[28] Das PM, Ramachandran K, vanWert J, Singal R. Chromatin immunoprecipitation assay. Biotechniques. 2004;37:961–9. 15597545 10.2144/04376RV01

[29] Zhao XC, Livingston MJ, Liang XL, Dong Z. Cell apoptosis and autophagy in renal fibrosis. Adv Exp Med Biol. 2019;1165:557–84. 31399985 10.1007/978-981-13-8871-2_28

[30] Teissier T, Quersin V, Gnemmi V, Daroux M, Howsam M, Delguste F, et al. Knockout of receptor for advanced glycation end-products attenuates age-related renal lesions. Aging Cell. 2019;18:e12850. 30794349 10.1111/acel.12850 PMC6413655

[31] Kerkeni M, Gharbi J. RAGE receptor: May be a potential inflammatory mediator for SARS-COV-2 infection? Med Hypotheses. 2020;144:109950. 32531537 10.1016/j.mehy.2020.109950 PMC7273142

[32] Yan SD, Chen X, Fu J, Chen M, Zhu H, Roher A, et al. RAGE and amyloid-beta peptide neurotoxicity in Alzheimer’s disease. Nature. 1996;382:685–91. 8751438 10.1038/382685a0

[33] Harcourt BE, Sourris KC, Coughlan MT, Walker KZ, Dougherty SL, Andrikopoulos S, et al. Targeted reduction of advanced glycation improves renal function in obesity. Kidney Int. 2011;80:190–8. 21412218 10.1038/ki.2011.57

[34] Matsunaga N, Anan I, Rosenberg P, Nagai R, Lundström O, Horiuchi S, et al. Advanced glycation end product is implicated in amyloid-related kidney complications. Scand J Clin Lab Invest. 2005;65:263–71. 16076681 10.1080/00365510510013794

[35] Fukami K, Yamagishi S, Ueda S, Okuda S. Role of AGEs in diabetic nephropathy. Curr Pharm Des. 2008;14:946–52. 18473844 10.2174/138161208784139710

[36] Tanji N, Markowitz GS, Fu C, Kislinger T, Taguchi A, Pischetsrieder M, et al. Expression of advanced glycation end products and their cellular receptor RAGE in diabetic nephropathy and nondiabetic renal disease. J Am Soc Nephrol. 2000;11:1656–66. 10966490 10.1681/ASN.V1191656

[37] Park EY, Seo MJ, Park JH. Effects of specific genes activating RAGE on polycystic kidney disease. Am J Nephrol. 2010;32:169–78. 20606421 10.1159/000315859

[38] D’Agati V, Schmidt AM. RAGE and the pathogenesis of chronic kidney disease. Nat Rev Nephrol. 2010;6:352–60. 20421886 10.1038/nrneph.2010.54

[39] Sadik NA, Mohamed WA, Ahmed MI. The association of receptor of advanced glycated end products and inflammatory mediators contributes to endothelial dysfunction in a prospective study of acute kidney injury patients with sepsis. Mol Cell Biochem. 2012;359:73–81. 21811803 10.1007/s11010-011-1001-4

