# Supplementary material for: Proximal tubular RAGE mediated the renal fibrosis in UUO model mice via upregulation of autophagy
Source: Cell Death Dis. 2022 Apr 23;13(4):399. doi: 10.1038/s41419-022-04856-z (PMC9035155; doi:10.1038/s41419-022-04856-z)
Supplement: Supplementary file 1 — supplementary [file 41419_2022_4856_MOESM1_ESM.docx]

# Proximal Tubular RAGE mediated the renal fibrosis in UUO model mice via up regulation of autophagy

Bohao Liu, Tianshi Sun, Huiling Li, Shuangfa Qiu ,Yijian Li, Xiaozhou Li, Peilin Zheng, Junxiang Chen, Xiangping Chai, Dongshan Zhang

Figure supplementary 1


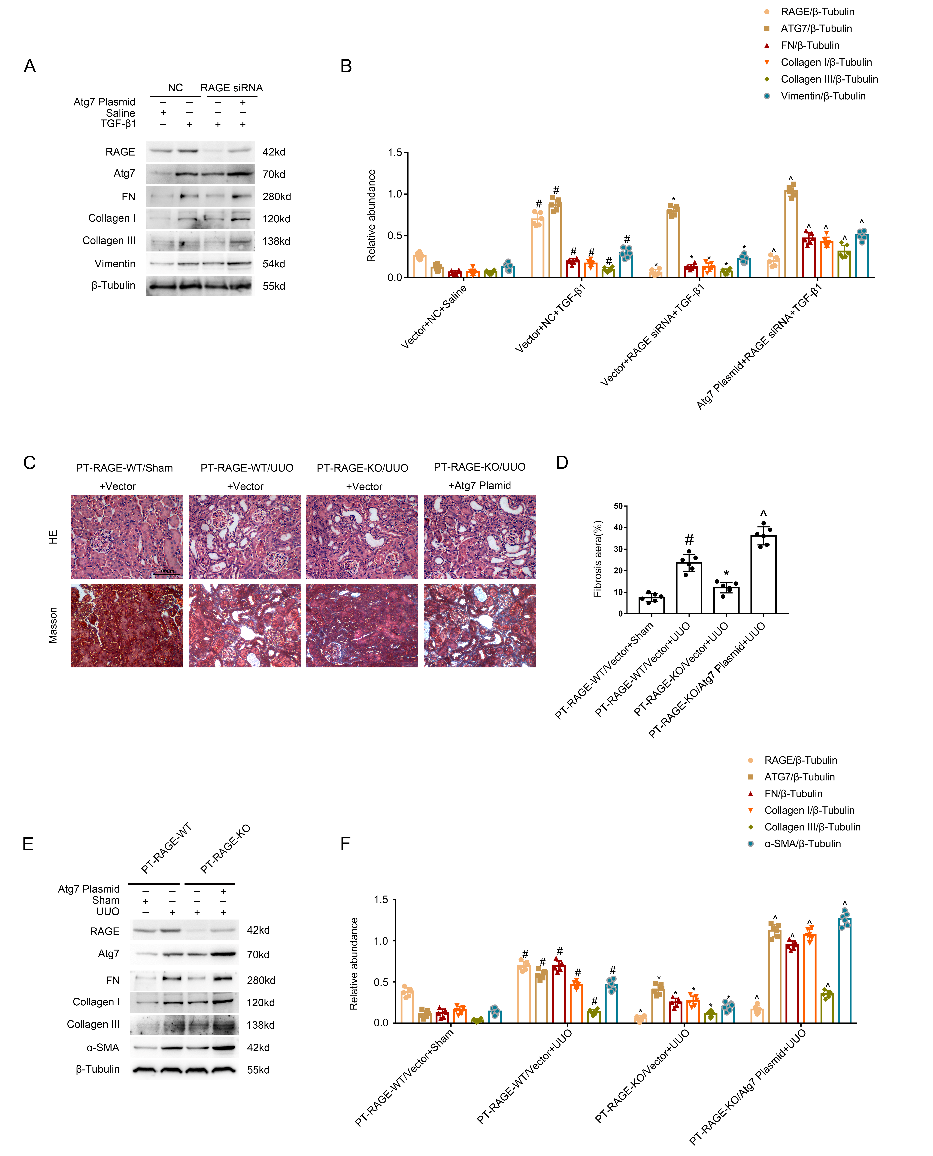


**Figure Supplementary 1. RAGE mediated TGF-β1-indced renal fibrosis was depended on the Atg7 in vitro and vivo .**

(**A,E)** Immunoblot analysis of RAGE & Atg7 & fibronectin & Col I&III, α-SMA and Vimentin. (**B,F)** Analysis of the grayscale image between them. **(C)** Hematoxylin and eosin staining and the representative Masson’s trichrome staining. (**D)** Quantification of tubulointerstitial fibrosis in the kidney cortex. Original magnification×400. Scale bar: 100 µM. Data are expressed as means ± s.d. (n = 6). #P < 0.05 versus Vector+NC+Saline or PT-RAGE-WT/Vector+Sham group. *P < 0.05 versus Vector+NC+TGF-β1 or PT-RAGE-WT/Vector+UUO group. ^P < 0.05 versus Vector+ RAGE siRNA + TGF-β1 or PT-RAGE-KO/Vector+UUO group. Each experiment (**A,C,E**) was repeated six times independently with similar results. (**B,D,F)** indicate the statistical Student’s t test used (means ± s.d., n = 6, P < 0.05)

Figure supplementary 2


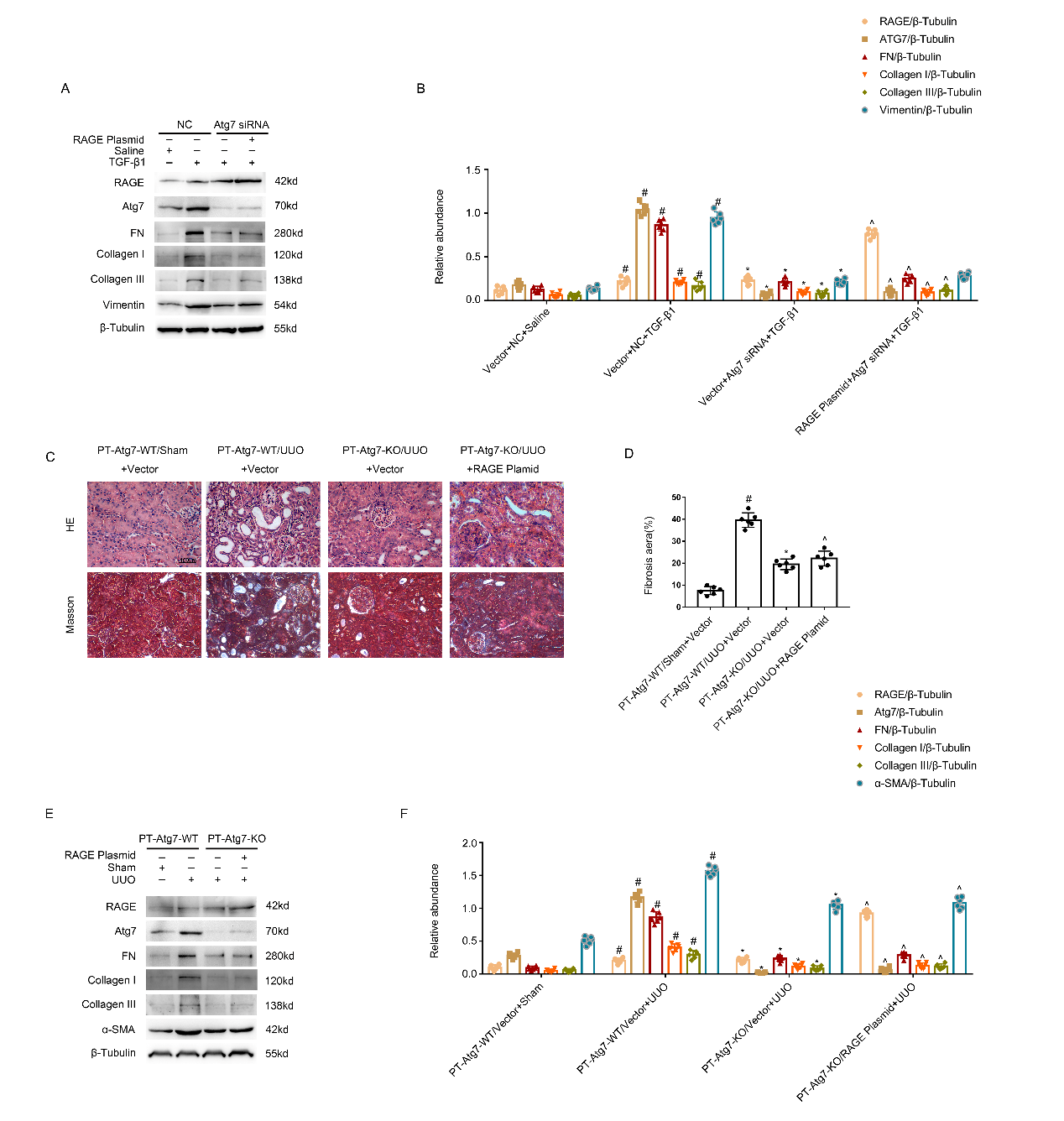


**Figure Supplementary 2.**

(**A,E)** Immunoblot analysis of RAGE & Atg7 & fibronectin & Col I&III, α-SMA and Vimentin. (**B,F)** Analysis of the grayscale image between them. **(C)** Hematoxylin and eosin staining and the representative Masson’s trichrome staining. (**D)** Quantification of tubulointerstitial fibrosis in the kidney cortex. Original magnification×400. Scale bar: 100 µM. Data are expressed as means ± s.d. (n = 6). #P < 0.05 versus Vector+NC+Saline or PT-ATG7-WT/Vector+Sham group. *P < 0.05 versus Vector+NC+TGF-β1 or PT-ATG7-WT/Vector+UUO group. ^P < 0.05 versus Vector+ Atg7 siRNA + TGF-β1 or PT-ATG7-KO/Vector+UUO group. Each experiment (**A,C,E**) was repeated six times independently with similar results. (**B,D,F)** indicate the statistical Student’s t test used (means ± s.d., n = 6, P < 0.05)

Figure supplementary 3


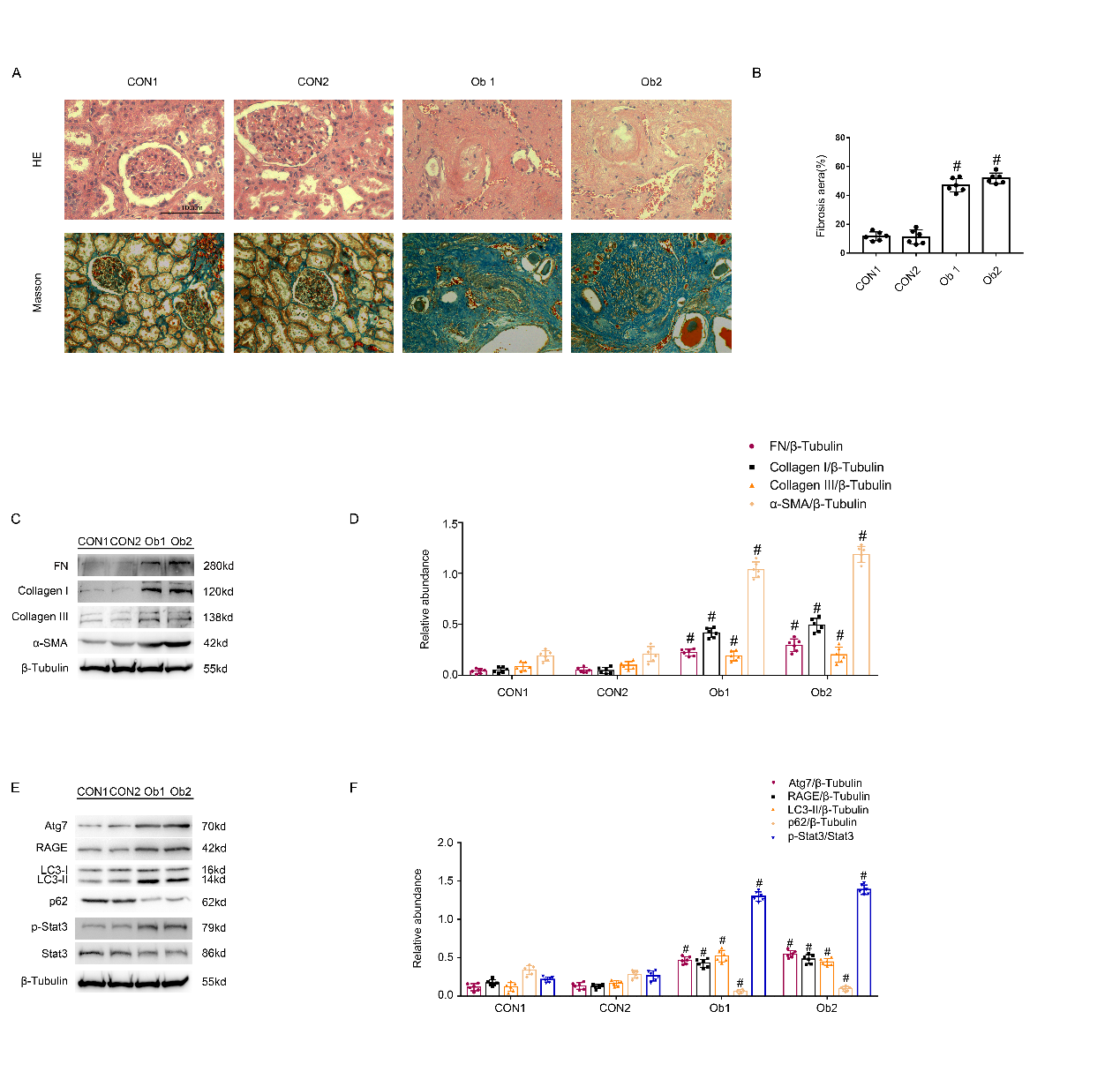
 **Figure Supplementary 3. Renal fibrosis and autophagy in the human kidneys.**

**(A)** Hematoxylin and eosin staining and the representative Masson’s trichrome staining. (**B)** Quantification of tubulointerstitial fibrosis in the kidney cortex. (**C,E)** Immunoblot analysis of fibronectin & Col I&III, α-SMA and LC3, p62. **(D,F)** Analysis of the grayscale image between them. Original magnification×400. Scale bar: 100 µM. Data are expressed as means ± s.d. (n = 6). #P < 0.05 versus CON group. Each experiment (**A,C,E**) was repeated six times independently with similar results. (**B,D,F)** indicate the statistical Student’s t test used (means ± s.d., n = 6, P < 0.05)

Figure supplementary 4


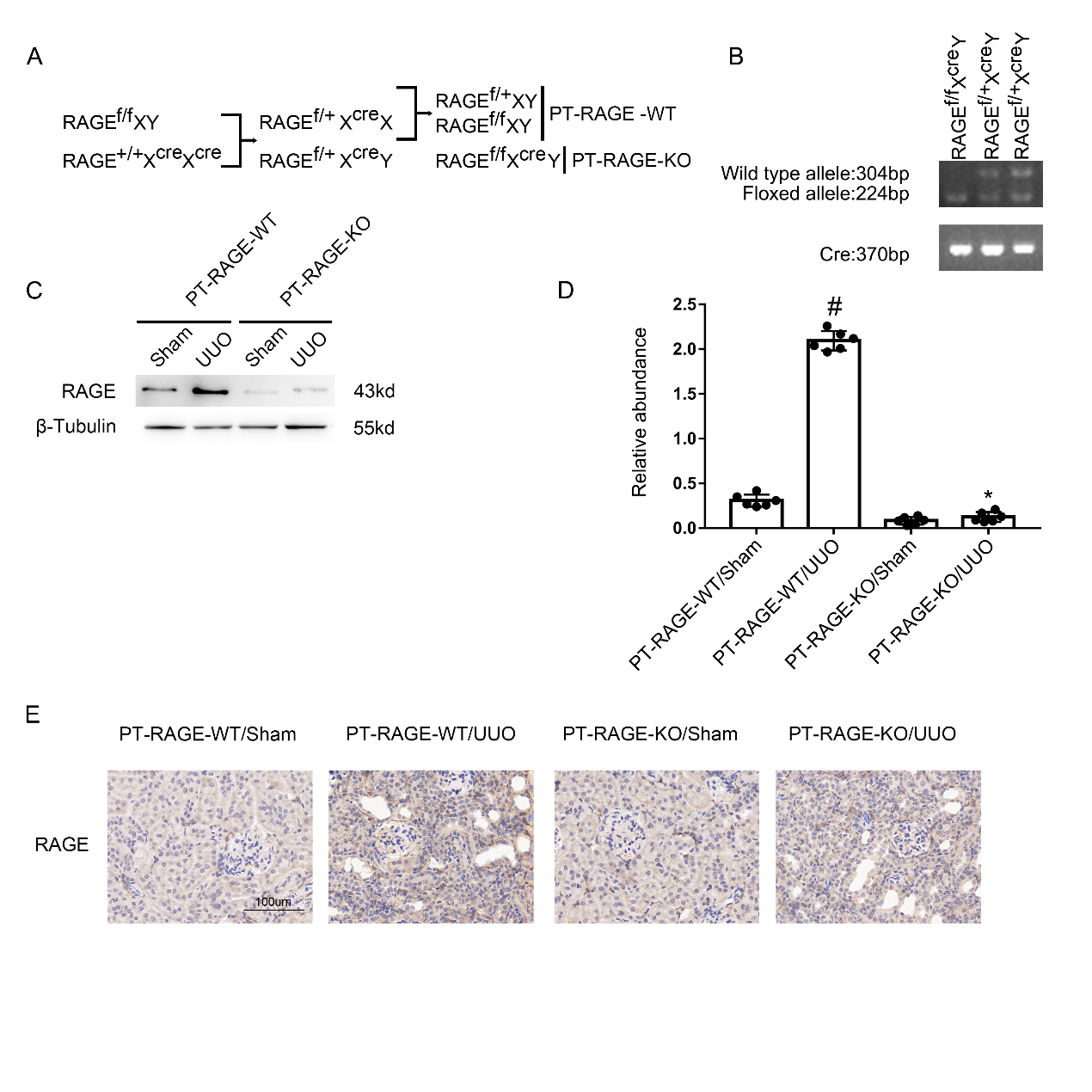


**Figure Supplementary 4. Generation and features of the PT-RAGE-KO mouse model.**

**(A)** Breeding procedure for the creation of PT-RAGE-KO mice. (**B)** PCR-based genotyping of wild-type and floxed alleles of RAGE and PEPCK-Cre allele. (**C)** Cortices and outer medulla of kidneys from PT-RAGE-KO and PT-RAGE-WT littermate mice following UUO injury were collected for immunoblot analysis of RAGE and β-Tubulin. (**D)** Analysis of the grayscale image between them. #P < 0.05 versus PT-RAGE-WT with the sham group. *P < 0.05 versus PT-RAGE-WT with UUO group. (**E)** Immunohistochemical staining of RAGE in the kidney cortical tissues of wild-type and RAGE-KO mice following UUO injury. Original magnification ×400. Scale bar: 100 µM. Data are expressed as means ± s.d. (n = 6). Each experiment (**C, E**) was repeated six times independently with similar results. (**D)** indicate the statistical Student’s t test used (means ± s.d., n = 6, P < 0.05).
