# Supplementary material for: Proximal tubular RAGE mediated the renal fibrosis in UUO model mice via upregulation of autophagy
Source: Cell Death Dis. 2022 Apr 23;13(4):399. doi: 10.1038/s41419-022-04856-z (PMC9035155; doi:10.1038/s41419-022-04856-z)
Supplement: Supplementary file 3 — Similarity Report [file 41419_2022_4856_MOESM3_ESM.pdf]

Manuscript

by Similarity Checker

Submission date: 30-Mar-2022 12:00AM (UTC-0700)
Submission ID: 1791866806
File name: Proximal_Tubular_RAGE_mediated.docx (105.78K)
Word count: 3192
Character count: 18741

Proximal Tubular RAGE mediated the kidney fibrosis in UUO model mice via up regulation of autophagy

Abstract

Previous studies reported that RAGE participated in the process of kidney fibrosis, but the function and regulation pathway of RAGE in proximal tubular cells in this process remains unclear. Here, we found that expression of RAGE was increased by TGF-β1 treatment and unilateral ureteral obstruction (UUO). Knock down of RAGE ameliorated renal fiborsis by TGF-β1 treatment, the expression of vimentin, Collagen I&III, and fibronectin are decreased. Mechanistically, RAGE mediated TGF-β1-induced phosphorylation of Stat3 and directly upregulated the Atg7 to increase the level of autophagy, and ultimately resulting in renal fibrosis. Furthermore, PT-RAGE-KO mice reduced kidney fibrosis in UUO model via inhibiting Stat3/Atg7 axis by knocking down RAGE. Furthermore, the above findings were confirmed in kidney of patients with obstructive nephropathy. Collectively, RAGE in proximal tubular cells promotes the autophagy to increase renal fibrosis via upregulation of Stat3/Atg7 axis.

Introduction

Chronic kidney disease (CKD) has a high prevalence rate[1,2], which seriously affects human health. It is estimated that CKD existed in about more than 10% of adults in developed countries[1]. Kidney fibrosis is a major pathological feature of CKD. The existing treatments for renal fibrosis are only slightly effective or ineffective[3]. Emerging data suggests that the tubular epithelium regulates renal fibrosis[2,4]. But mechanisms of tubular epithelium in renal fibrosis are still poorly understood.

Receptor for advanced glycation end products (RAGE) regulates the innate immune response via binding of numerous exogenous and endogenous ligands [5]. Recent studies report that it not only plays a pivotal role in early tissue repair in disease[6], but also is increased in the procession of occurrence and development of a variety diseases including cancer, diabetes, neurodegeneration[7].

Advanced glycation end products (AGEs) directly bind to RAGE, and leads to the increasing of expression level of cytokines and growth factors, including vascular endothelial growth factor and connective tissue growth factor, finally results in glomerular injury[8-13]. Besides, previous study reported that global RAGE knockout mice reduced renal interstitial fibrosis via downregulation of transforming growth factor (TGF)-β[14]. But the function and mechanism of proximal tubular RAGE in renal fibrosis remain unclear according to available data.

Autophagy is a process which cytoplasmic components are degraded by lysosomes[15], The role of autophagy is associated with the type of cell or tissue and the experimental model[16]. For example, UUO-induced kidney fibrosis was exacerbated in PT-ATG5-KO autophagy deficiency mice or LC3(-/-) mice (deletion of LC3B)[17,18] , this was alleviated in PT-ATG7-KO autophagy deficiency mice in contrary [17]. The data indicated that roles of autophagy in UUO-kidney fibrosis remain controversial. In addition, AGEs bind to RAGE and then induces autophagy in various diseases including heart disease and colorectal cancer[19-22], which suggested that RAGE partly mediates autophagy production. But the function and pathway of proximal tubular RAGE in UUO-induced autophagy are still unclear. According to the above literature, we hypothesize that proximal tubular RAGE can induce kidney fibrosis by regulating autophagy.

In our present study, we observed that RAGE was induced by UUO and TGF-β1, and then mediated autophagy to increase the renal fibrosis via STAT3/Atg7 axis *in vitro and vivo*. These results contribute to our understanding of the renoprotection by proximal tubular RAGE deletion in response to UUO.

Methods

Antibodies and reagents.

Anti-β-Tubulin (ab175186), anti-RAGE (ab216329), anti-fibronectin (ab2413), anti-Collagen I (ab138492), anti-Collagen III (ab184993), anti-vimentin (ab92547), and anti-α-SMA (ab124964) antibodies were purchased from Abcam (Cambridge, UK). Anti-Stat3 (9139), Phospho-Stat3 (4074), Atg7 (2631), and LC3-I/II (4108) were supplied by Cell Signaling Technology (MA, USA), while anti-p62,SQSTM1 (Cat No.18420-1-AP) were obtained from Proteintech (IL, USA).

Recombinant human TGF-β1 (7754-BH) was obtained from R&D Systems (MN, USA). The RAGE siRNA and Atg7 siRNA were supplied by Santa Cruz Biotechnology. Atg7 and GFP-LC3-I/II plasmid was constructed by the Ruqi company(Guanzhou, China).

Animal experiments

The proximal tubule-specific RAGE or Atg7-deletion mice were produced by crossing RAGE or Atg7 (flox/flox) mice (obtained from Dr Wang Lab and Dr Xiong Lab, respectively) with PEPCK-Cre mice. The UUO model was constructed by ligating the left ureter in mice[2]. After obtaining ethical approval, the animals were experimented in compliance with the guidelines approved by the Animal Care Ethics Committee of Second Xiangya Hospital, China. The mice were housed at stable room temperature in a 12/12-h light/dark cycle and accessed to standard rodent chow and water.

Human samples

The project was approved by the Review Board of Second Xiangya Hospital, China, kidney biopsy samples were collected from obstructive nephropathy (Ob) patients as Ob group (n = 8) and normal subjects as control group (n = 8). We announce that all experiments were performed in compliance with the Declaration of Helsinki principles, and also complied with the guidance of the Ministry of Science and Technology for the Review and Approval of Human Genetic Resources. The inclusion criteria of Ob were referred to a previous study[2].

Cell culture, transfection, and treatment

BUMPT cells were grown in DMEM medium (Thermo-Fisher-Scientific) containing 10% FBS and antibiotics in a 37 °C incubator with 5% CO_2. BUMPT cells were tansfected with plasmids or siRNA using lipofection 2000 for twenty-four hours, followed by overnight starvation in a serum-free medium. Subsequently, the cells were treated with/without 5 ng/ml TGF-β1 for another 24 h. Bovine serum albumin (0.1%) was used as control.

Histological, immunohistochemical, immunofluorescence, and Western blot analyses.

Renal tissues were collected and stained with HE and Masson's trichrome as previous described[2] . Immunohistochemical analysis was carried out using anti-RAGE (1:50), α-SMA (1:100), Collagen

I/III (1:100) and fibronectin (1:100) in accordance with the previous instruction[2]. The immunofluorescence of puncta was performed following the standard process. Whole BUMPT cell or kidney tissue protein lysates were separated through SDS-PAGE, and then tranfered to membrane and subsequently exposed to primary antibodies of RAGE, STAT3, p-STAT3, α-SMA, Collagen I/III, fibronectin, vimentin, and Atg7 following by the second antibody according to the standard procedure.

Establishment of PT-RAGE-KO mice

To explore the role of proximal tubular RAGE in renal fibrosis, we constructed a proximal tubules of RAGE-KO mouse model. The breeding instruction is denoted in FigS4 A, floxed RAGE alleles in male mice (RAGE$^{f/f}$ XY) were crossed with female phosphoenolpyruvate carboxy kinase-cAMP-response element (PEPCK-Cre) transgenic mice (RAGE$^{+/+}$X^{cre}X$^{cre)}$. After the first-generation born, heterozygous female offsprings (RAGE$^{f/+}$X^{cre}X) were crossed with RAGE$^{f/+}$X^{cre}Y male mice to generate the proximal tubule RAGE wild-type (PT-RAGE-WT) and PT-RAGE-KO (RAGE$^{f/f}$X^{cre}Y) littermate mice. To identify genotypes, each mouse was subjected to three sets of PCR. PT-RAGE-KO mice has three genotypic features: (i) the 224-bp DNA fragment floxed allele is amplified; (ii) the 304-bp DNA fragment WT allele is insufficiently amplified; (iii) the 370-bp DNA fragment in Cre gene is amplified (FigS4 B). Western blot analysis revealed that the RAGE expression level in the renal cortices of PT-RAGE-KO mice was decreased compared to PT-RAGE-WT under sham and UUO injury treatment (see FigS4 C&D), this was further confirmed by the immunohistochemistry result (see FigS4 E). The data indicated that tubular RAGE was knockdown in this conditional KO model.

ChIP assay

Chromatin immunoprecipitation (ChIP) assay was performed using commercial kit (Millipore, MA, USA) with primary antibodies against STAT3[23]. The following primer pairs were employed to analyze the precipitated DNA through RT-qPCR: SBS1: 5′-CTGGCGGGGTTCA CTTAGGA-3′ and 5′-TCACAGTCCGGGGACACAAG-3′; SBS2: 5′-CCGAATCAACCTGCG TCTGC-3′, and 5′-CGGCCTCTGCTTCACTGAGT-3′; SBS3: 5′-TGTTGTTGTAGTGCGC ATGC-3′, and

5′-GGGGACCGAGTATAGACAGGT-3′; SBS4:5′-GGCCACGGAGTAAGCTTGT G-3′, and 5′-TGTGTCCGATTGCCTAGGCT-3′.

Statistics.

Quantitave data were described as mean ± standard deviation. Student’s t test was employed for comparing two groups, whereas one-way ANOVA was employed for comparing multiple groups. Level of statistical significance was set at $P<0.05$.

Results

Expression of RAGE is upregulated by TGF-β1 in BUMPT cells, UUO in mice, and in the renal cortices of Ob-patients.

At the outset, the expression of RAGE in obstructive nephropathy (Ob) patients was upregulated significantly compared to the control group (Fig. 1C&F). Next, this founding was also confirmed in BUMPTs treated with/without TGF-β1 (Fig. 1A&D), which was the same as the results observed in the kidney tissues of UUO or sham mice (Fig. 1B&E). Moreover, the immunohistochemistry staining of RAGE verified our above results (Fig. 1G). RAGE expression was induced by TGF-β1 in BUMPTs, UUO in mice, and in the renal cortices of Ob-patients.

RAGE mediates the TGF-β1-stimulated expression of fibronectin, vimentin and Collagen I&III in BUMPTs.

To determine the role of RAGE in renal fibrosis, BUMPTs were first transfected with RAGE siRNA or RAGE plasmid, and then administered with/without TGF-β1. The immunoblot results showed that TGF-β1-stimulated the enhancement of expression levels of fibronectin (FN), Collagen I&III, vimentin, and RAGE was attenuated by RAGE siRNA (see Fig. 2A-F), by the contrast, these changes were improved by the overexpression of RAGE plasmids (see Fig. 2 G–L). The data demonstrated that RAGE at least partly mediated TGF-β1-stimulated the expression

of fibronectin, vimentin and Collagen I&III.

RAGE mediated the TGF-β1-induced autophagy in BUMPTs.

Previous research has demonstrated that RAGE mediated AGEs induced autophagy, but the role of RAGE in TGF-β1-triggered autophagy remains unclear. The immunoblot analysis demonstrated that TGF-β1-stimulated the upregulation of RAGE and LC3 II, and the reduction of p62 was markedly ameliorated by RAGE siRNA (see Fig. 3A&C), well, this was augmented by RAGE plasmids (see Fig. 3B&D). To observed autophagosome formation, GFP-LC3 was used to transfect HK-2 cells, which given a granular, punctate stain in the cytoplasm. As shown in Fig. 3E&F, the LC3 puncta numbers and intensity both improved after the treatment of TGF-β1, which was reduced by RAGE siRNA but augmented by RAGE plasmids. This finding was consistent with the immunoblot results. The data showed that RAGE mediated TGF-β1 induced autophagy.

The RAGE mediated the TGF-β1-induced autophagy in BUMPT cells via STAT3/Atg7 axis

The above finding revealed that RAGE promoted the renal fibrosis, however, the regulation mechanism remains largely unknown. In current study, the immunoblotting results showed that TGF-β1-stimulated enhancement of p-Stat3, Atg7, and LC3 II, and the reduction of p62 was notably attenuated by STAT3-IN-1, an STAT3 inhibitor (see Fig. 4A&B). Furthermore, we observed that the administration of STAT3-IN-1 reduced the mean number of LC3 puncta in each cell vs TGF-β1 group (see Fig. 4C&D). Then, we investigated whether STAT3 is responsible for the upregulation of Atg7 during TGF-β1-induced autophagy in BUMPT cells. The predication result of JASPAR CORE database (http://jaspar.Genereg.net/) indicated that Atg7 promotor sequence contain four binding sites of STAT3. ChIP assays showed a binding sites (a 227-bp fragment) for STAT3 in the in promoter region of Atg7 (see Fig. 4E). Finally, the immunobloting results showed that TGF-β1-induced the Stat3/Atg7 signal pathway was attenuated by the RAGE siRNA (see Fig. 4F&H), oppositely, this was notably enhanced by the RAGE plasmid (see Fig. 4F&H). Collectively, the data revealed that RAGE/STAT3/Atg7 axis could mediate TGF-β1-triggered autophagy in BUMPT cells.

Atg7 mediates TGF-β1-stimulated expression of fibronectin, vimentin and Collagen I&III in BUMPT cells.

The association between autophagy and renal fibrosis remains controversy[24]. UUO-induced kidney fibrosis was exacerbated in PT-ATG5-KO autophagy deficiency mice or LC3(-/-) mice(deletion of LC3B)[17,18] , in contrary, this was ameliorated in PT-ATG7-KO autophagy deficiency mice[17] Here, we focused on the Atg7**, a core autophagy-related protein.** BUMPT cells transfected with Atg7 siRNA or Atg7 plasmid were treated with TGF-B1 as well as control vehicle. As displayed in Fig. 4A&B, TGF-β1-stimulated the increasing of Atg7, LC3 II, fibronectin, Collagen I&III, and vimentin as well as the reduction of p62 was markedly suppressed by the Atg7 siRNA (see Fig.5A&B), by the contrast, this was reinforced by the Atg7 plasmid (see Fig.5C&D). We further verified that TGF-β1-stimulated the enhancement of the mean number of LC3 puncta/cell was attenuated by the Atg7 siRNA, however, this was augmented by the Atg7 plasmid (see Fig.5E&F). The data suggest that autophagy is at least partly responsible for the increasing of fibronectin, vimentin and Collagen I&III induced by the TGF-β1.

UUO-induced renal fibrosis was ameliorated in PT-ATG7-KO mice

In order to confirm the role of Atg7 in renal fibrosis, PT-ATG7-KO mice was established, and then subjected to UUO model for 7 days. The results of HE and Masson staining demonstrated that UUO-induced tubular damage and renal interstitium fibrosis was ameliorated in PT-ATG7-KO mice (see Fig. 6A-B). Then, we performed immunochemical staining which showed that the expression of α-SMA, Collagen I&III, and fibronectin was upregulated in UUO model of PT-ATG7-WT mice, whereas it was notably ameliorated in PT-ATG7-KO mice (see Fig. 6C&D). The immunoblotting analysis revealed that UUO-induced the enhancement of Atg7, LC3 II, α-SMA, Collagen I&III and fibronectin, and the reduction of p62 was markedly decreased in PT-ATG7-KO mice (see Fig. 7A–H). The data verified that Atg7 plays an essential role in UUO-induced renal fibrosis.

UUO-induced kidney fibrosis is alleviated in PT-RAGE-KO mice via inhibition of STAT3/Atg7 axis

The PT-RAGE-KO and PT-RAGE-WT littermate mice were subjected to UUO model for 7 days. The results of HE and Masson staining demonstrated that UUO-induced tubular damage and renal interstitium fibrosis was alleviated in PT-RAGE-KO mice (see Fig. 8A-B). The data of immunochemical staining showed that UUO-induced the expression of α-SMA, Collagen I&III, and fibronectin was remarkably decreased in PT-RAGE-KO mice (Fig. 8 C&D). The immunoblotting analysis demonstrated that UUO-induced induced the increasing of α-SMA, Collagen I&III, fibronectin, Atg7, LC3 II as well as the reduction of p62 was markedly reversed in PT-RAGE-KO mice (Fig. 8 E–H). The data confirmed that Proximal Tubular RAGE mediated the renal fibrosis in UUO model mice via up regulation of autophagy.

RAGE mediates TGF-β1-indced kidney fibrosis depended on the Atg7 *in vitro and vivo*

To further explore whether RAGE promotes the renal fibrosis via autophagy during TGF-β1 treatment, we carried out the following experiments. Firstly, RAGE siRNA significantly ameliorated the TGF-β1-stimulated the expression of α-SMA, Collagen I&III, and fibronectin in BUMPTs, which was markedly reversed by the overexpression of Atg7 (see FigS1, E&F). Consistently, the results of HE and Masson staining demonstrated that PT-RAGE-KO attenuated UUO-induced tubular damage and renal interstitium fibrosis, which was attenuated by the injection of Atg7 plasmid (see FigS1, A&B). The Western blot data showed that PT-RAGE-KO mice not only suppressed the UUO-induced kidney fibrosis but also the expression of α-SMA, Col I&III, and fibronectin, which confirmed the above pathological results (see FigS1, C&D). Secondly, the data of HE and Masson staining revealed that UUO-induced tubular damage and renal interstitium fibrosis was alleviated in PT-Atg7-KO mice which was injected with RAGE Plasmid (see FigS2, A&B). Atg7 siRNA remarkably decreased the TGF-β1-stimulated the expression of α-SMA, Collagen I&III, and fibronectin in BUMPTs, this was not reversed by the overexpression of RAGE (see FigS2, E&F). Consistently, PT-Atg7-KO mice not only reduced the UUO-induced kidney fibrosis but also the expression of α-SMA, Col I&III, and fibronectin, this was also not reversed by the overexpression of RAGE (see FigS2, C&D). The data demonstrated that RAGE induced the renal fibrosis depended on the autophagy *in vitro and vivo* models.

RAGE/Stat3/Atg7 axis mediated renal fibrosis in patients with Ob.

The data of HE and Masson staining showed that patients with Ob-triggered tubular damage and renal interstitium fibrosis than the patients with MCD (see Fig. S3 A&B). Immunoblot data also revealed that patients with Ob-induced the expression of α-SMA, Collagen I&III, and fibronectin, Atg7, RAGE, LC3II, and p-stat3 as well as the decreasing of p62 than the patients with MCD (see FigS3 C-F).

Discussion

Previous studies demonstrated that global RAGE knock out attenuated that renal fibrosis[14,25]. This study for the first time demonstrated that proximal tubular RAGE also attenuated the kidney fibrosis induced by TGF-β1 *in vitro* and UUO *in vivo*. Mechanistically, RAGE mediated the TGF-β1-triggered Stat3 activation and then promoted autophagy to increase renal fibrosis via upregulation of Atg7. The data suggested that tubular RAGE may be considered as a therapy target.

Several studies have shown that the AGEs-RAGE pathway plays vital roles in the progression of various kidney disorders including hypertensive nephropathy, diabetic nephropathy, lupus nephritis, obesity-related glomerulopathy, amyloidosis, ADPKD, and septic AKI[26-33]. The global knock out or inhibition of RAGE were used to block the AGEs-RAGE pathway in above studies. Hence, the role of proximal tubular RAGE remains Unclarified. In the current study, we firstly assessed the increasing of RAGE in the kidneys of Ob patients and UUO mice likewise in TGF-β1-treated BUMPTs (see Fig. 1). Secondly, we demonstrated that tubular RAGE mediated the renal fibrosis, which was supported by the following evidences: 1) RAGE siRNA ameliorated TGF-β1-stimulated the expression of vimentin, collagen I&III, and fibronectin (see Fig.2). 2) PT-RAGE-KO mice notably ameliorated UUO-induced renal fibrosis (see Fig 9).

Previous research has reported that RAGE could regulate the formation of autophagy. For example, Dr Gao et al reported that RAGE mediated the autophagy via upregulation of pp65-NF

κB and BNIP3[19] in pressure overload-induced heart failure. Dr Hou et al found that RAGE mediated AGEs-induced autophagy in cardiomyocyte injury via PI3K/AKT/mTOR pathway[20]. Dr Huang et al demonstrated that RAGE promoted the autophagy in colorectal cancer via ERK/Drp1 phosphorylation[21]. Dr Meng et al verified that RAGE promoted the autophagy in diabetes-associated osteoporosis through Raf/MEK/ERK signaling pathway[22]. In present study, we found that RAGE mediated autophagy, which was demonstrated by the following findings:1) TGF-β1-triggered autophagy was attenuated by the RAGE siRNA, which was improved by the overexpression of RAGE (see Fig.3). 2) PT-RAGE-KO mice notably ameliorated the UUO-induced autophagy (see Fig. 9). Furthermore, we found that Stat3/Atg7 signal pathway is responsible for the autophagy production mediated by tubular RAGE during TGF-β1 and UUO treatment. The following evidences supported the above finding: 1) Inactivation of Stat3 signal pathway reduced TGF-β1 induced autophagy via downregulation of Atg7 (see Fig4. A-D). 2) ChIP analysis for the first time indicated that Stat3 directly binds the promoter of Atg7(see Fig4. E). 3) The Stat3/Atg7 signal pathway was suppressed by the RAGE siRNA or PT-RAGE-KO (see Fig4. F-I and Fig.9. E-I), by the contrast, this was improved by RAGE overexpression. In addition, this signal pathway was also upregulated expressed in OB patients (see Fig.S3). Together, these results support that RAGE/Stat3/Atg7 axis mediated TGF-β1 and UUO-induced autophagy.

The roles of autophagy in renal fibrosis still need to be clarified. The research from two groups reported that PT-ATG5-KO autophagy deficiency or global knock out of LC3B exacerbated UUO-induced renal fibrosis[17,18]. However, Dr Dong at al found that PT-ATG7-KO autophagy deficiency mice attenuated UUO-induced renal fibrosis[17]. In this study, we found that PT-ATG7-KO autophagy deficiency mice notably reduced the renal fibrosis in UUO model (see Fig.6&7). In addition, TGF-β1-stimulated the expression of vimentin, collagen I&III, and fibronectin was notably reduced by the Atg7 knockdown, by contrast, this was augmented by the overexpression of Atg7 (see Fig.5). Our results are consistent with the Dr Dong's finding. Finally, we found that overexpression of Atg7 diminished the protective role of RAGE siRNA or PT-RAGE-KO on kidney fibrosis induced by TGF-β1 or UUO (see FigS1), however, the protective role of Atg7 siRNA or PT-ATG7-KO on kidney fibrosis induced by TGF-β1 or UUO was not enhanced by the overexpression of RAGE. The data suggested that RAGE promoted

autophagy to exacerbate renal fibrosis during TGF-β1 or UUO treatment.

In conclusion, we found that proximal tabular RAGE-mediated renal fibrosis *in vitro and vivo*. Mechanistically, TGF-β1 stimulated the RAGE and then activated STAT3 to increase autophagy via directly upregulation of Atg7, and then promoted the progression of renal fibrosis. Our study suggests that the RAGE/STAT3/Atg7 axis can serve as a therapeutic target of kidney fibrosis.

Manuscript

ORIGINALITY REPORT

14%	9%	13%	1%
SIMILARITY INDEX	INTERNET SOURCES	PUBLICATIONS	STUDENT PAPERS

PRIMARY SOURCES

1	www.ncbi.nlm.nih.gov<br>Internet Source	2%
2	Kai Ai, Lei Yi, Yinhuai Wang. "CircRNA_33702 promotes renal fibrosis by targeting the miR-29b-3p/WISP1 pathway", Cold Spring Harbor Laboratory, 2022<br>Publication	2%
3	Xiaozhou Li, Jian Pan, Huiling Li, Guangdi Li et al. "DsbA-L mediated renal tubulointerstitial fibrosis in UUO mice", Nature Communications, 2020<br>Publication	2%
4	www.asn-online.org<br>Internet Source	1%
5	"Renal Fibrosis: Mechanisms and Therapies", Springer Science and Business Media LLC, 2019<br>Publication	1%
6	Kai Ai, Jian Pan, Pan Zhang, Huiling Li et al. "Methyl-CpG-binding domain protein 2 contributes to renal fibrosis through	1%

promoting polarized M1 macrophages", Cell Death & Disease, 2022
Publication

7 Dongshan Zhang, Yu Liu, Qingqing Wei, Yuqing Huo, Kebin Liu, Fuyou Liu, Zheng Dong. "Tubular p53 Regulates Multiple Genes to Mediate AKI", Journal of the American Society of Nephrology, 2014 — 1%
Publication

8 www.nature.com — 1%
Internet Source

9 Man J. Livingston, Han-Fei Ding, Shuang Huang, Joseph A. Hill, Xiao-Ming Yin, Zheng Dong. "Persistent activation of autophagy in kidney tubular cells promotes renal interstitial fibrosis during unilateral ureteral obstruction", Autophagy, 2016 — 1%
Publication

10 Xuan Xu, Jian Pan, Huiling Li, Xiaozhou Li, Fang Fang, Dengke Wu, Yu Zhou, Peiling Zheng, Li Xiong, Dongshan Zhang. "Atg7 mediates renal tubular cell apoptosis in vancomycin nephrotoxicity through activation of PKC‐δ", The FASEB Journal, 2018 — 1%
Publication

11 cyberleninka.org — <1%
Internet Source

12	thno.org<br>Internet Source	<1 %
13	www.aging-us.com<br>Internet Source	<1 %
14	www.jove.com<br>Internet Source	<1 %
15	Jie Duan, Xishi Liu, Honglin Wang, Sun-Wei Guo. "The M2a macrophage subset may be critically involved in the fibrogenesis of endometriosis in mice", Reproductive BioMedicine Online, 2018<br>Publication	<1 %
16	Www.semanticscholar.org<br>Internet Source	<1 %
17	www.freepatentsonline.com<br>Internet Source	<1 %
18	afritradomedic.com<br>Internet Source	<1 %
19	www.science.gov<br>Internet Source	<1 %
20	www.spandidos-publications.com<br>Internet Source	<1 %
21	Chang Chen, Jingya Gu, Jue Wang, Yu Wu, Aihua Yang, Tingting Chen, Tingting Zhou, Zhaoguo Liu. "Physcion 8-O-β-	<1 %

glucopyranoside ameliorates liver fibrosis through inflammation inhibition by regulating SIRT3-mediated NF-κB P65 nuclear expression", International Immunopharmacology, 2021
Publication

22 Yong-Heng Luo, Ping-Bo Ouyang, Jiao Tian, Xiao-Jian Guo, Xuan-Chu Duan. "Rosiglitazone Inhibits TGF-β 1 Induced Activation of Human Tenon Fibroblasts via p38 Signal Pathway", PLoS ONE, 2014
Publication
<1%

23 Jiang, Man, Qingqing Wei, Guie Dong, Masaaki Komatsu, Yunchao Su, and Zheng Dong. "Autophagy in proximal tubules protects against acute kidney injury", Kidney International, 2012.
Publication
<1%

24 Li-Juan Liu, Feng-Juan Yao, Gui-Hua Lu, Cheng-Gui Xu, Zhe Xu, Kai Tang, Yun-Jiu Cheng, Xiu-Ren Gao, Su-Hua Wu. "The Role of the Rho/ROCK Pathway in Ang II and TGF-β1-Induced Atrial Remodeling", PLOS ONE, 2016
Publication
<1%

Exclude quotes	Off	Exclude matches	Off
Exclude bibliography	Off		
