# Supplementary material for: Proximal tubular RAGE mediated the renal fibrosis in UUO model mice via upregulation of autophagy
Source: Cell Death Dis. 2022 Apr 23;13(4):399. doi: 10.1038/s41419-022-04856-z (PMC9035155; doi:10.1038/s41419-022-04856-z)
Supplement: Supplementary file 4 — Original Data File [file 41419_2022_4856_MOESM4_ESM.pdf]

Figure1

RAGE

β-Tubulin

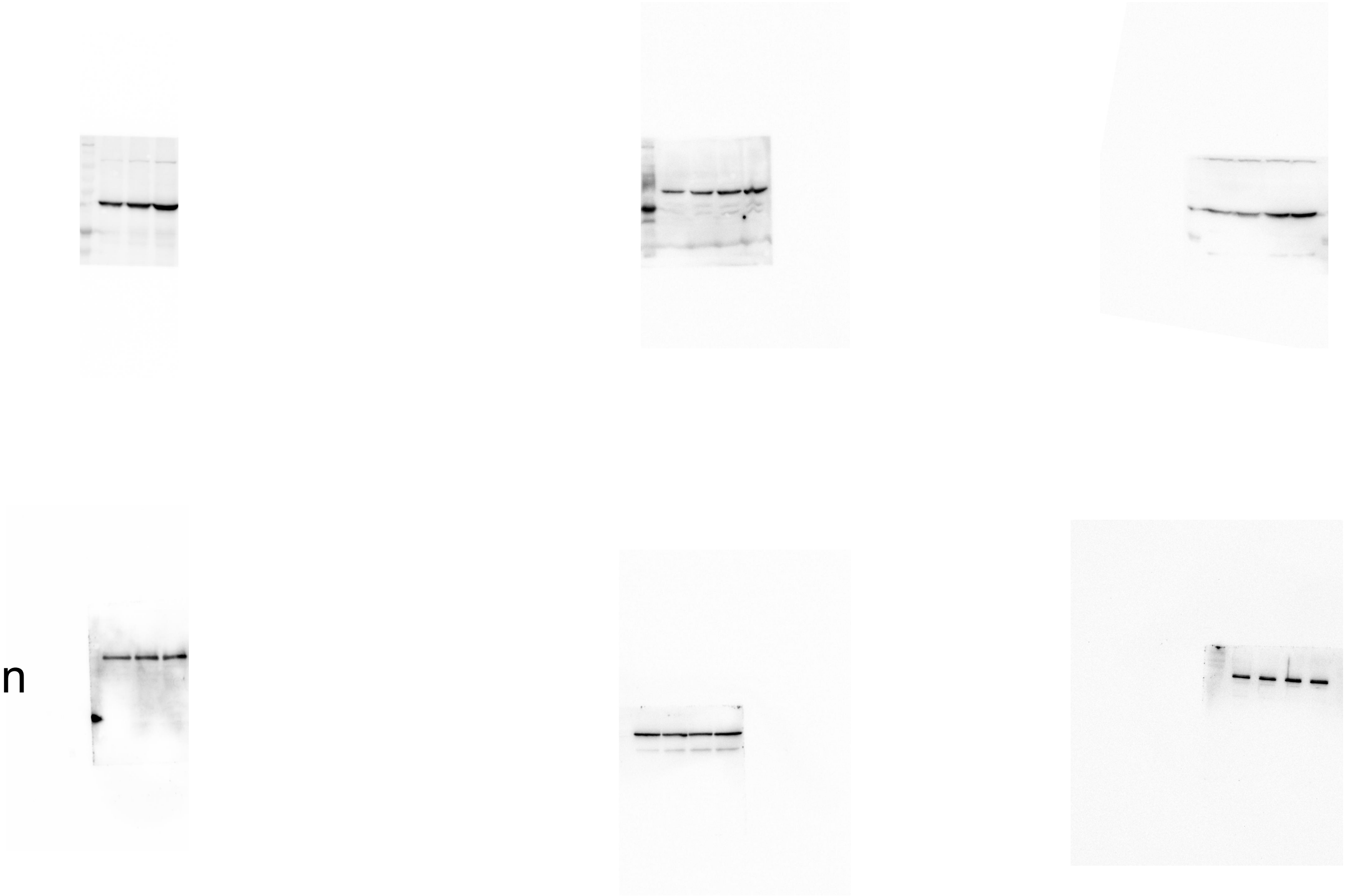

Figure1

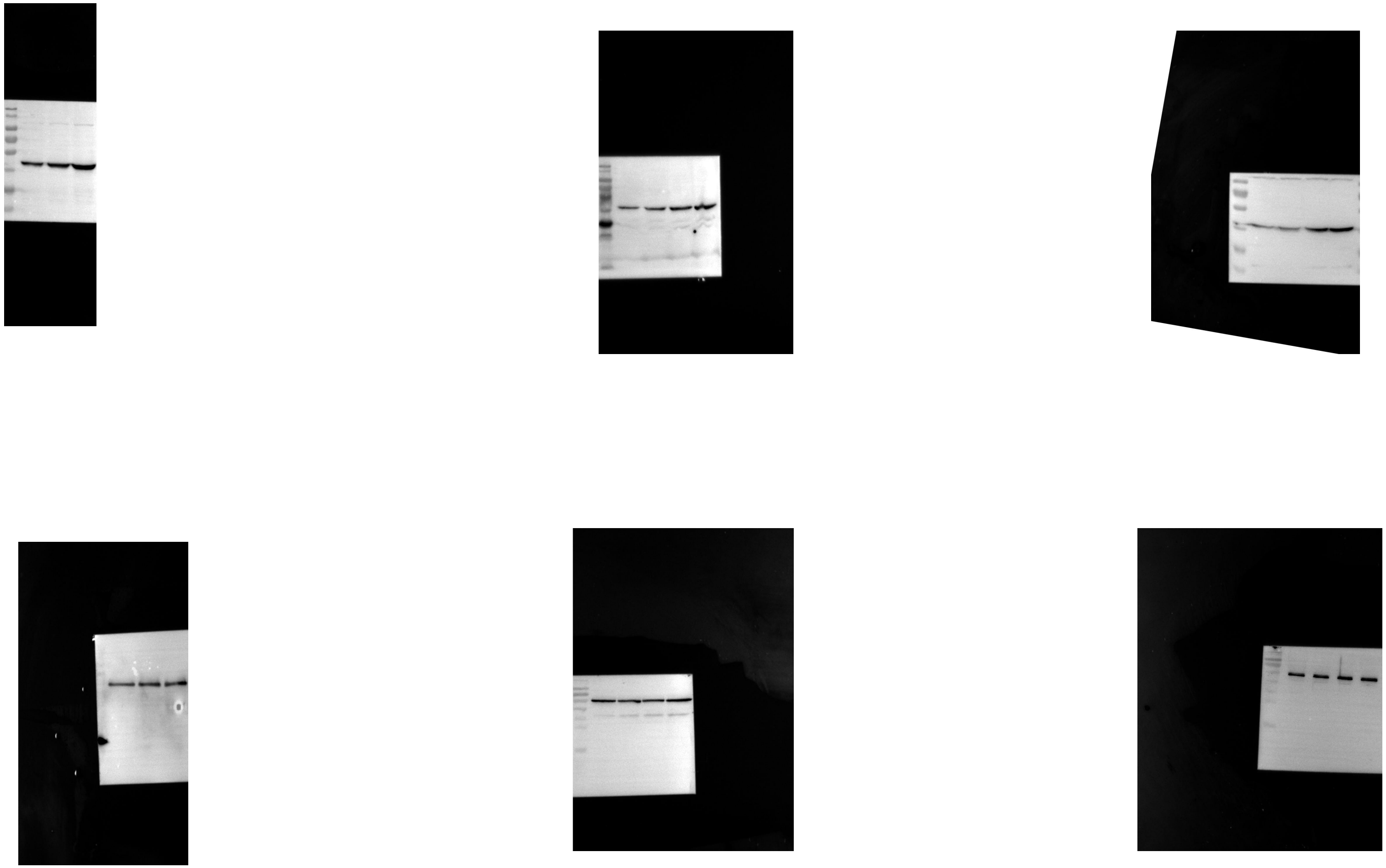

Figure2

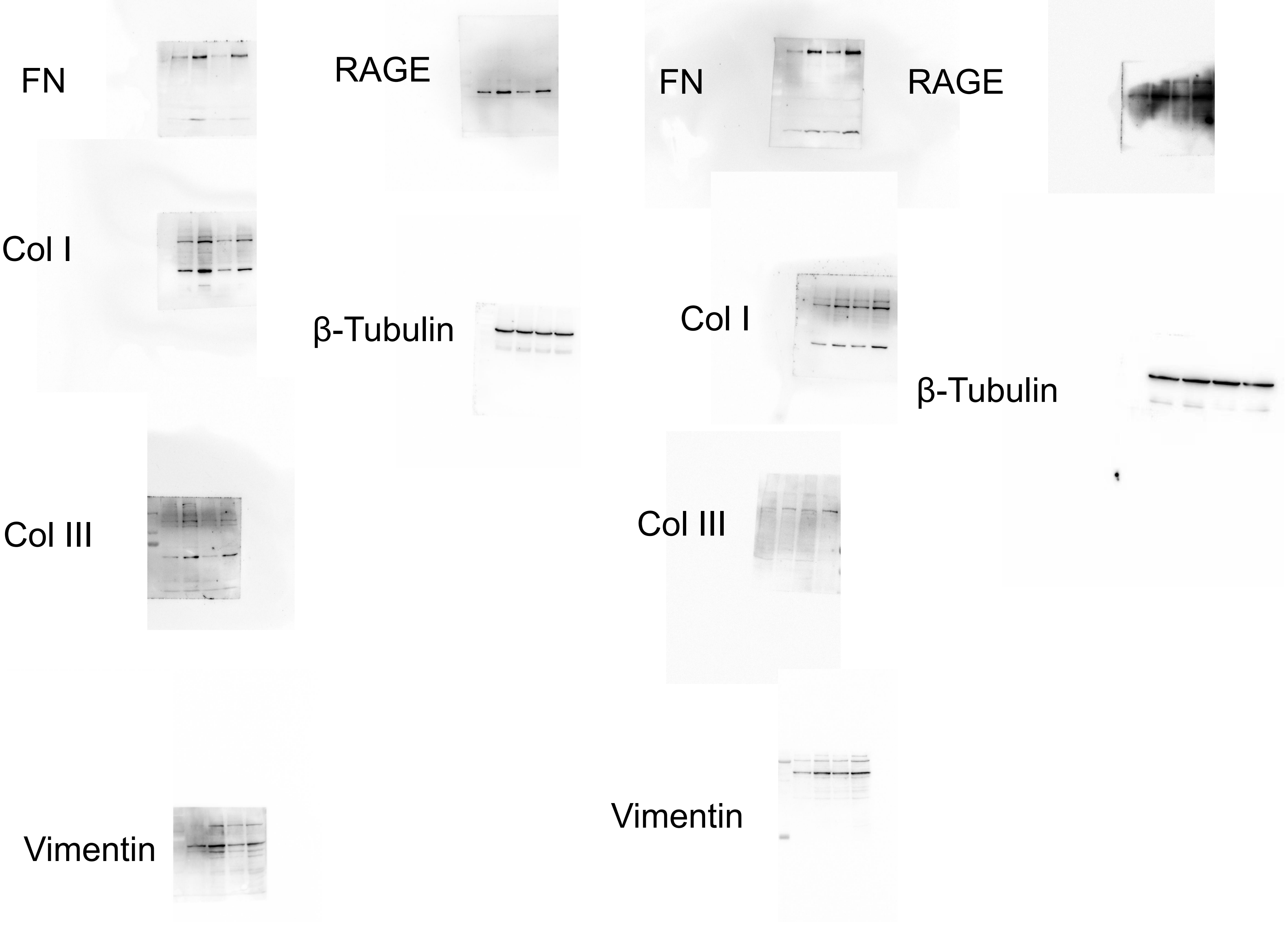
FN
RAGE
FN
RAGE
Col I
β-Tubulin
Col I
β-Tubulin
Col III
Col III
Vimentin
Vimentin

Figure2

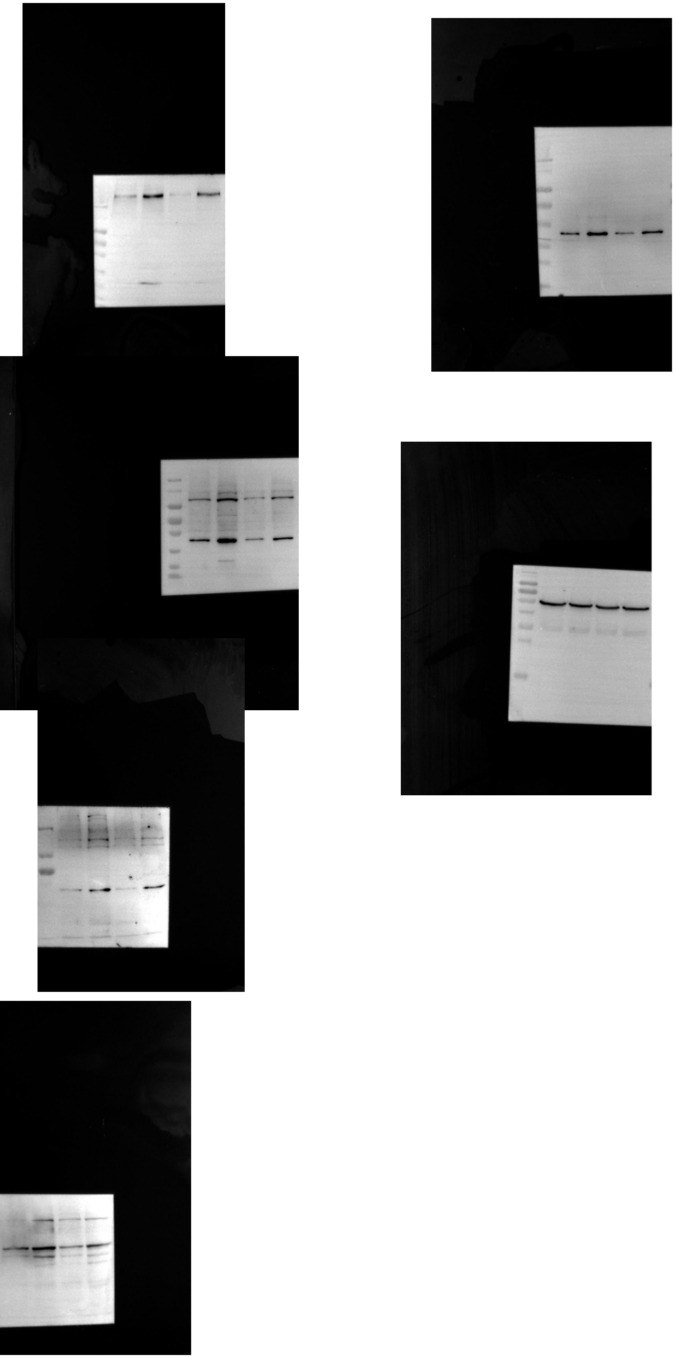

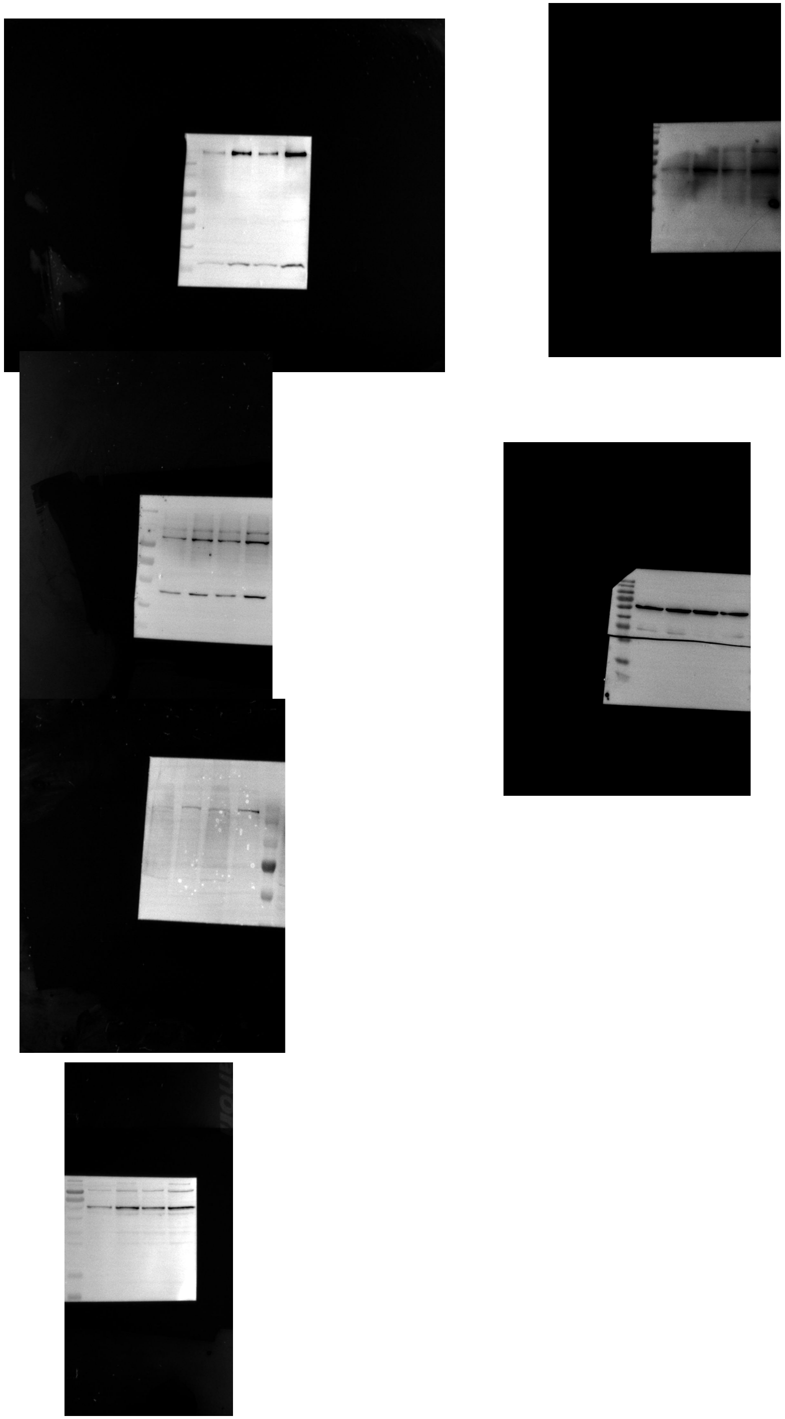

Figure3

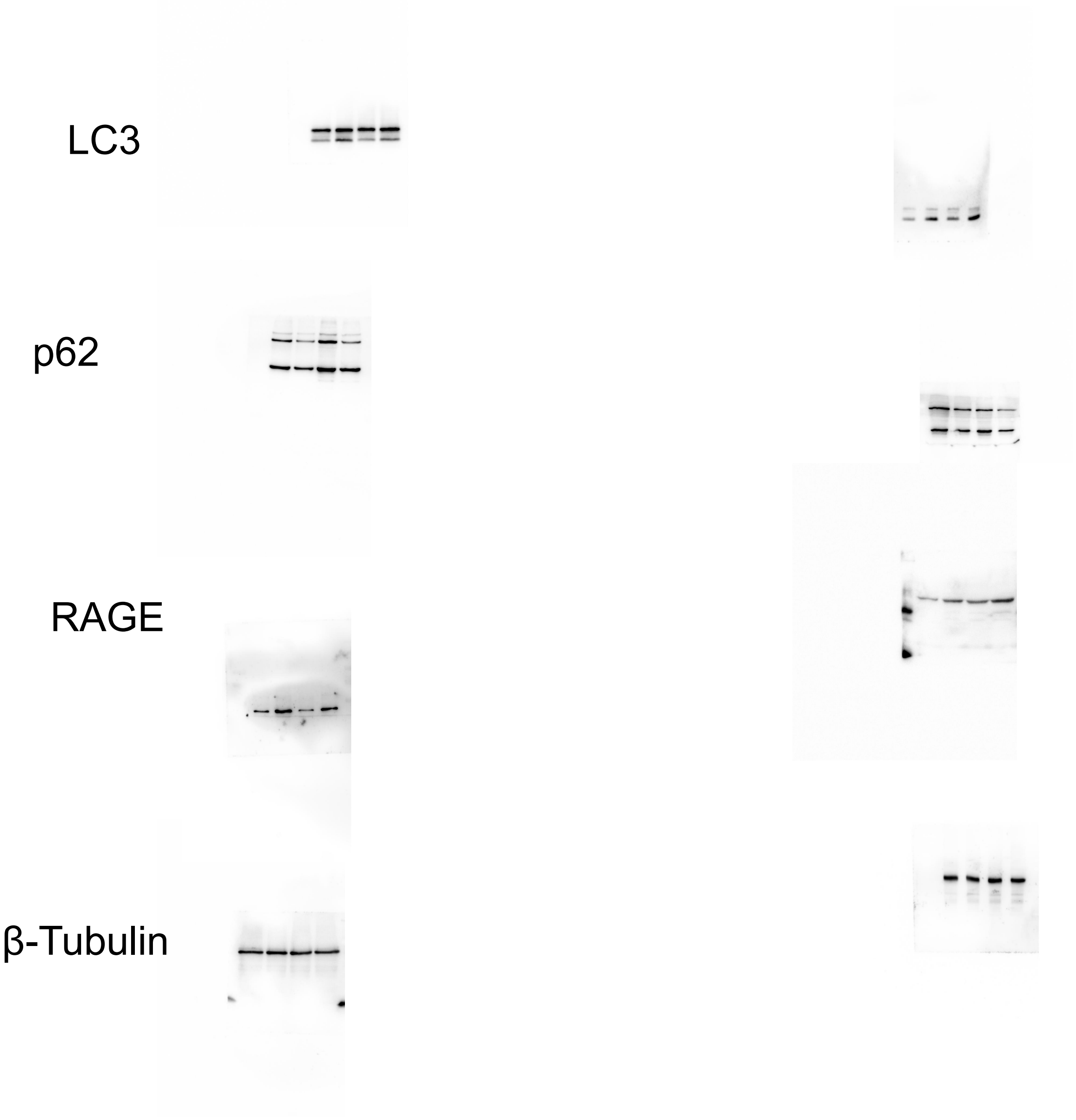

Figure3

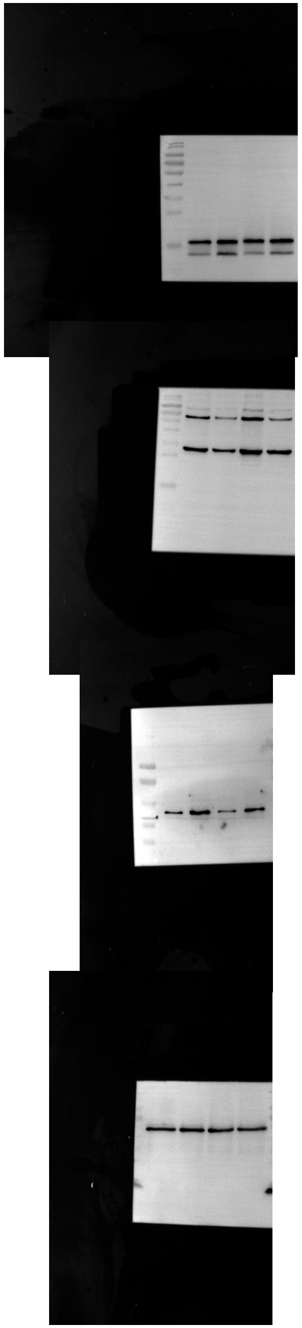

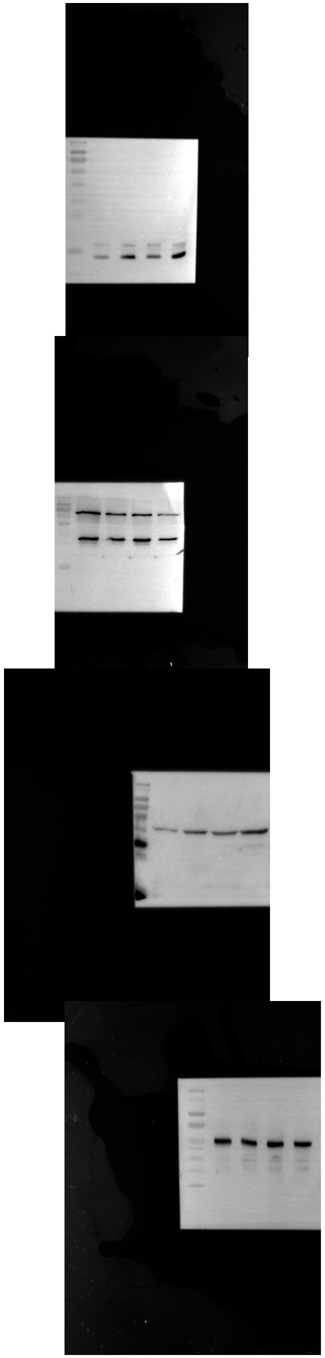

Figure4

LC3

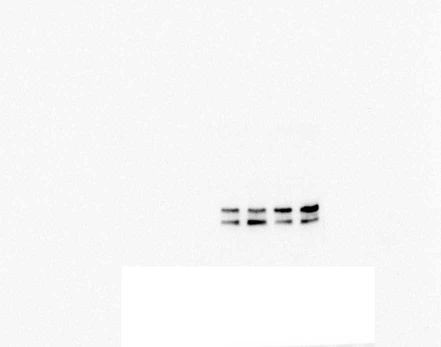

p62

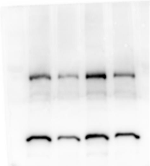

Atg7

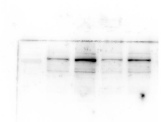

p-Stat3

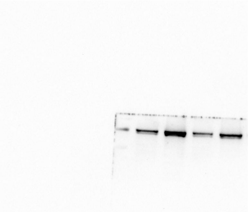

Stat3

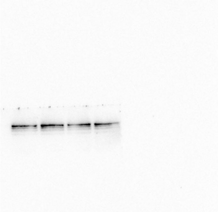

β-Tubulin

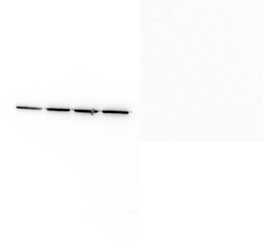

Stat3

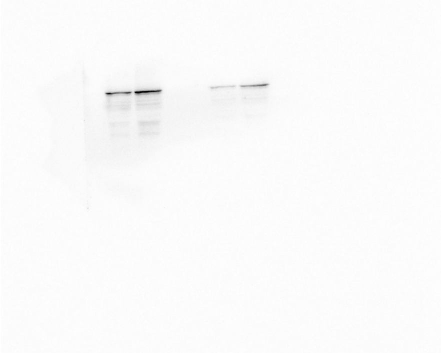

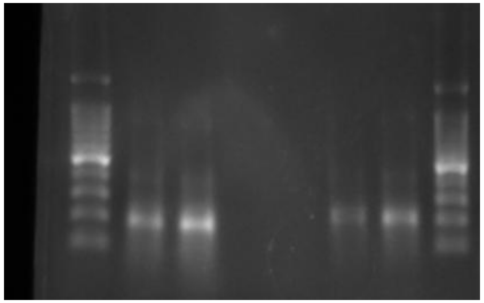

Figure4

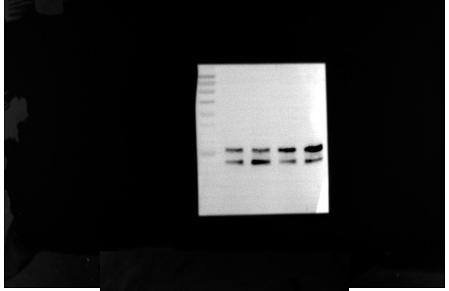

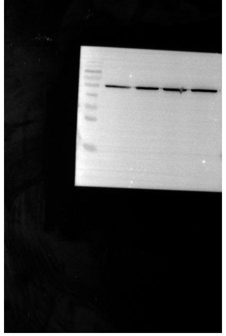

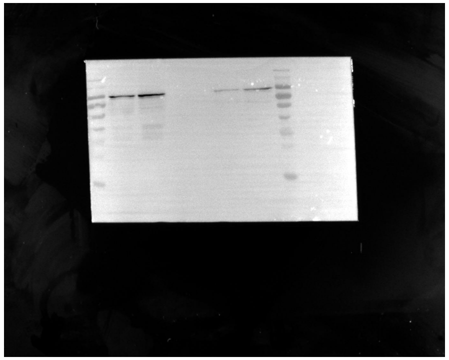

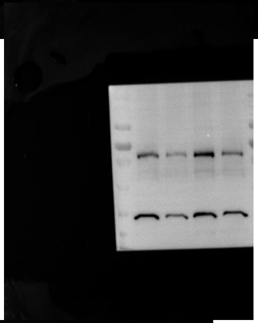

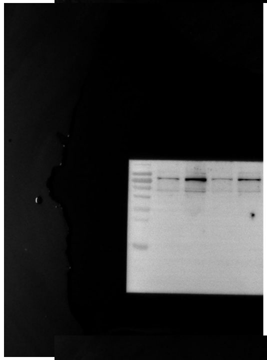

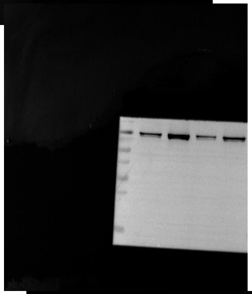

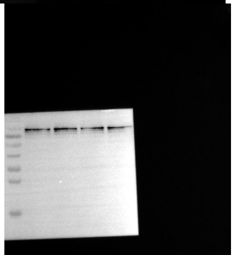

Figure5

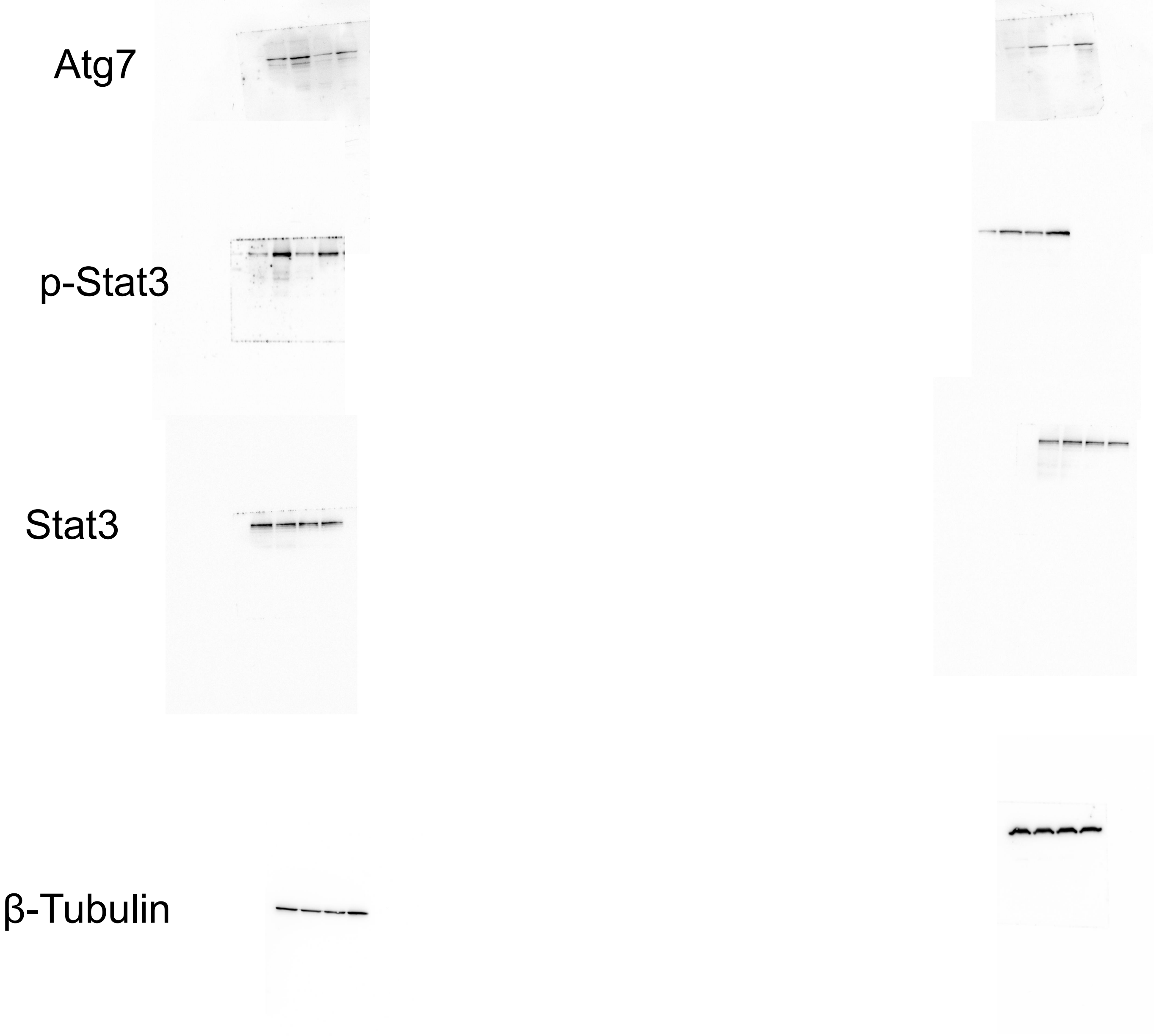

Figure5

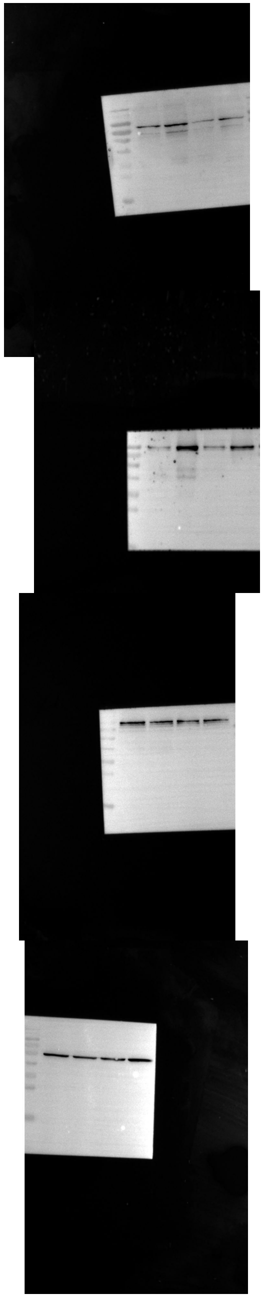

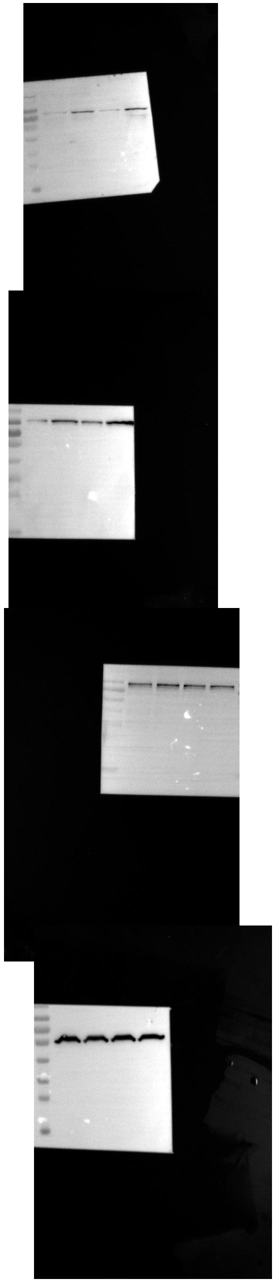

Figure6

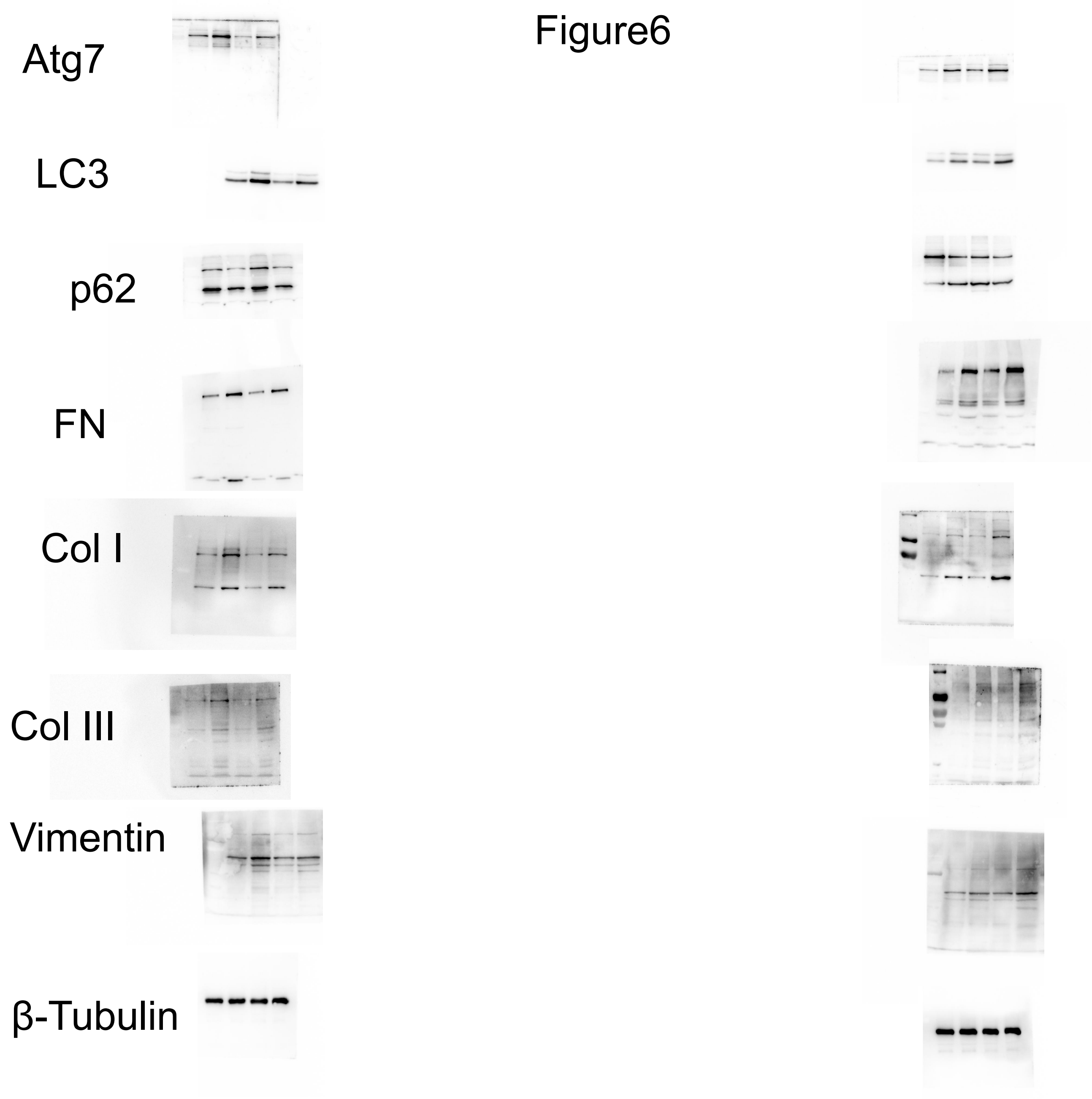

Figure6

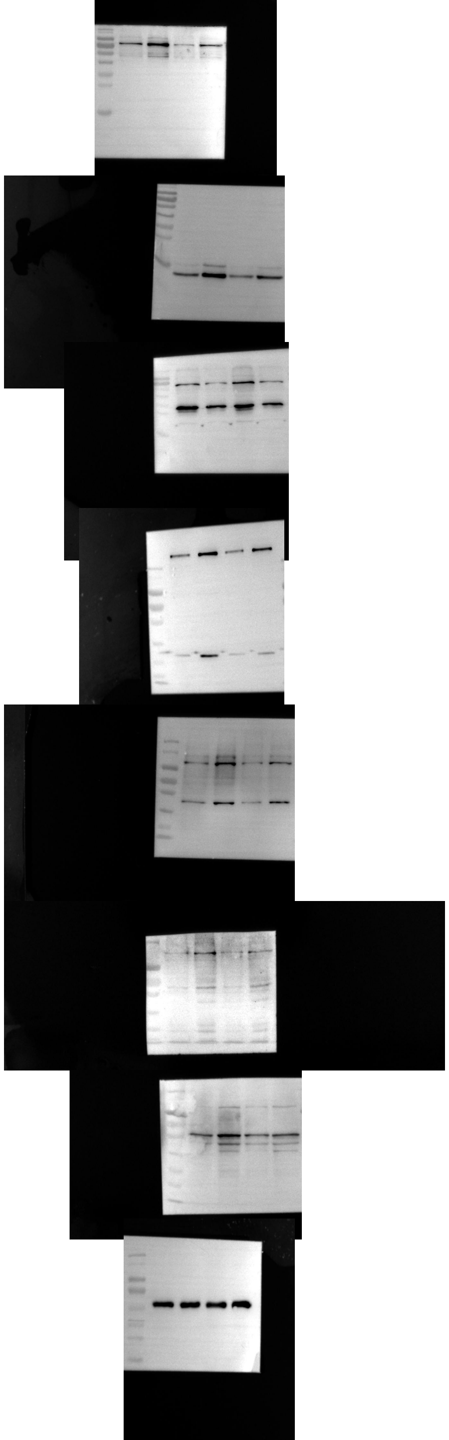

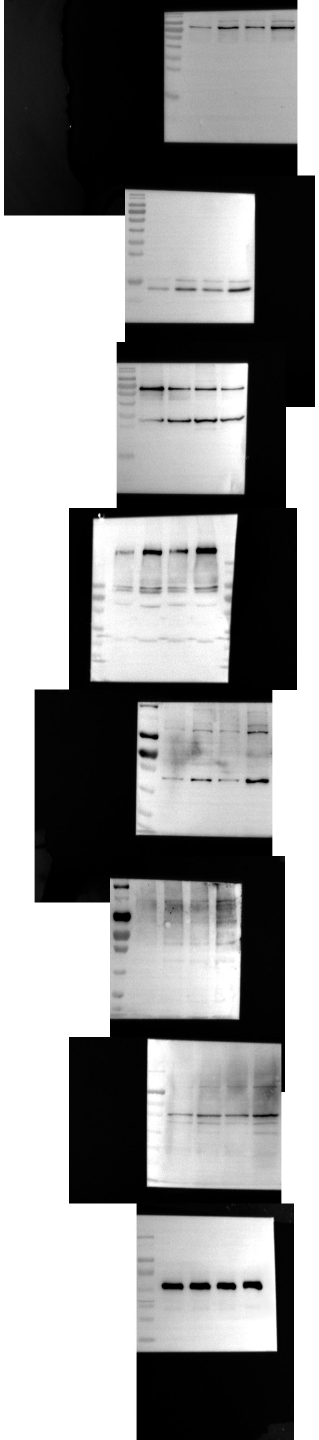

Figure7

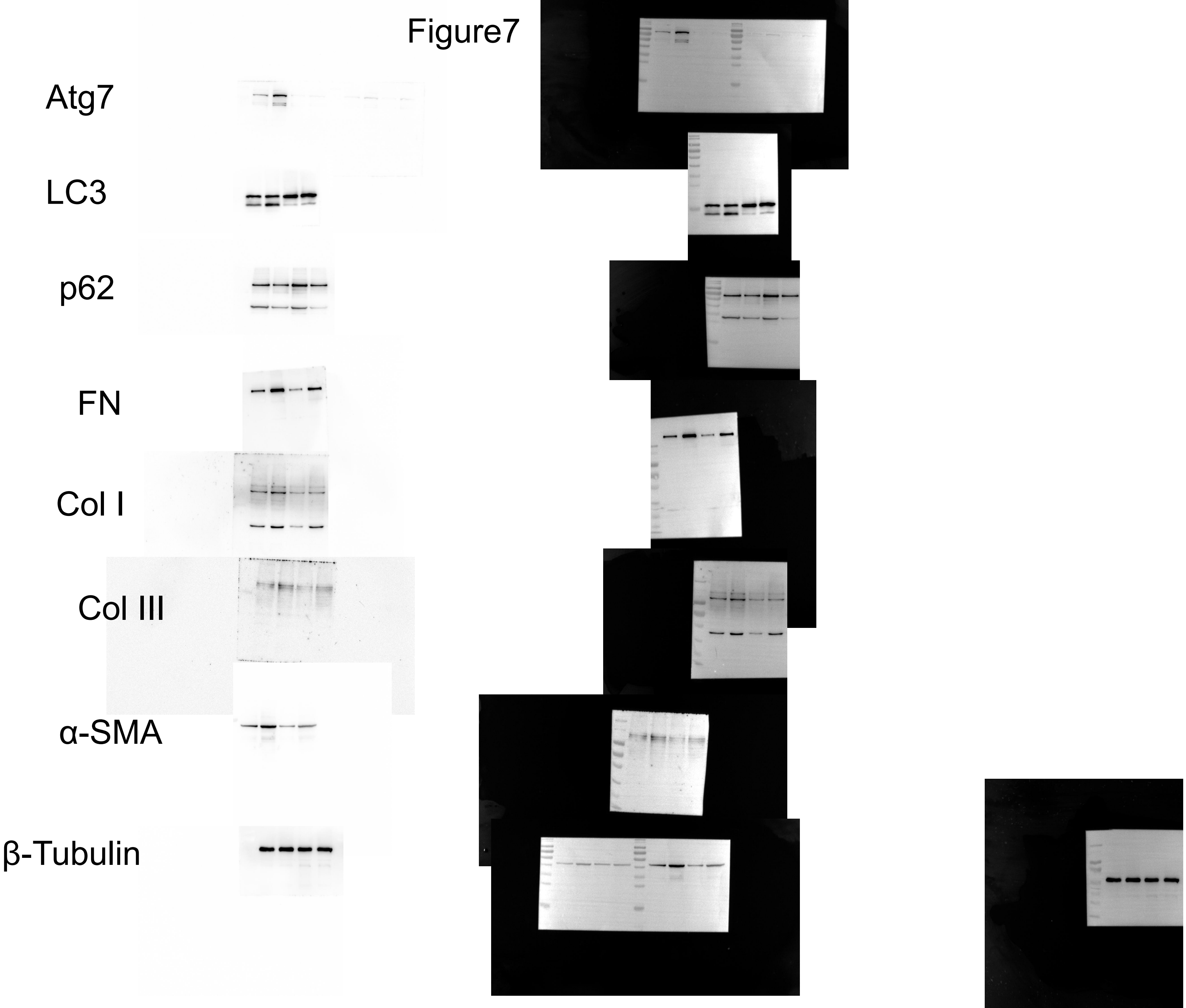

Figure8

RAGE

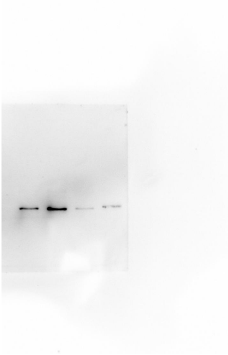

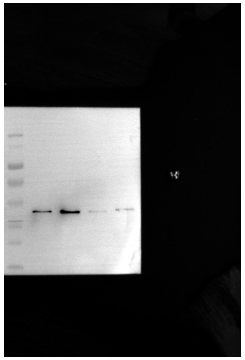

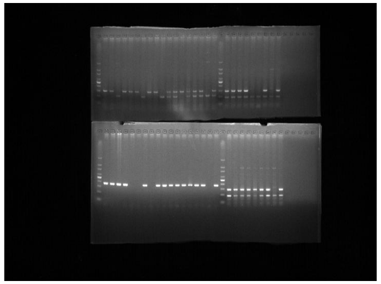

β-Tubulin

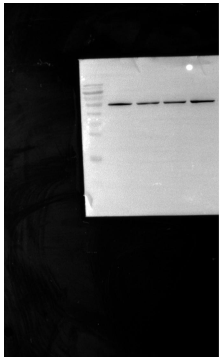

Figure9

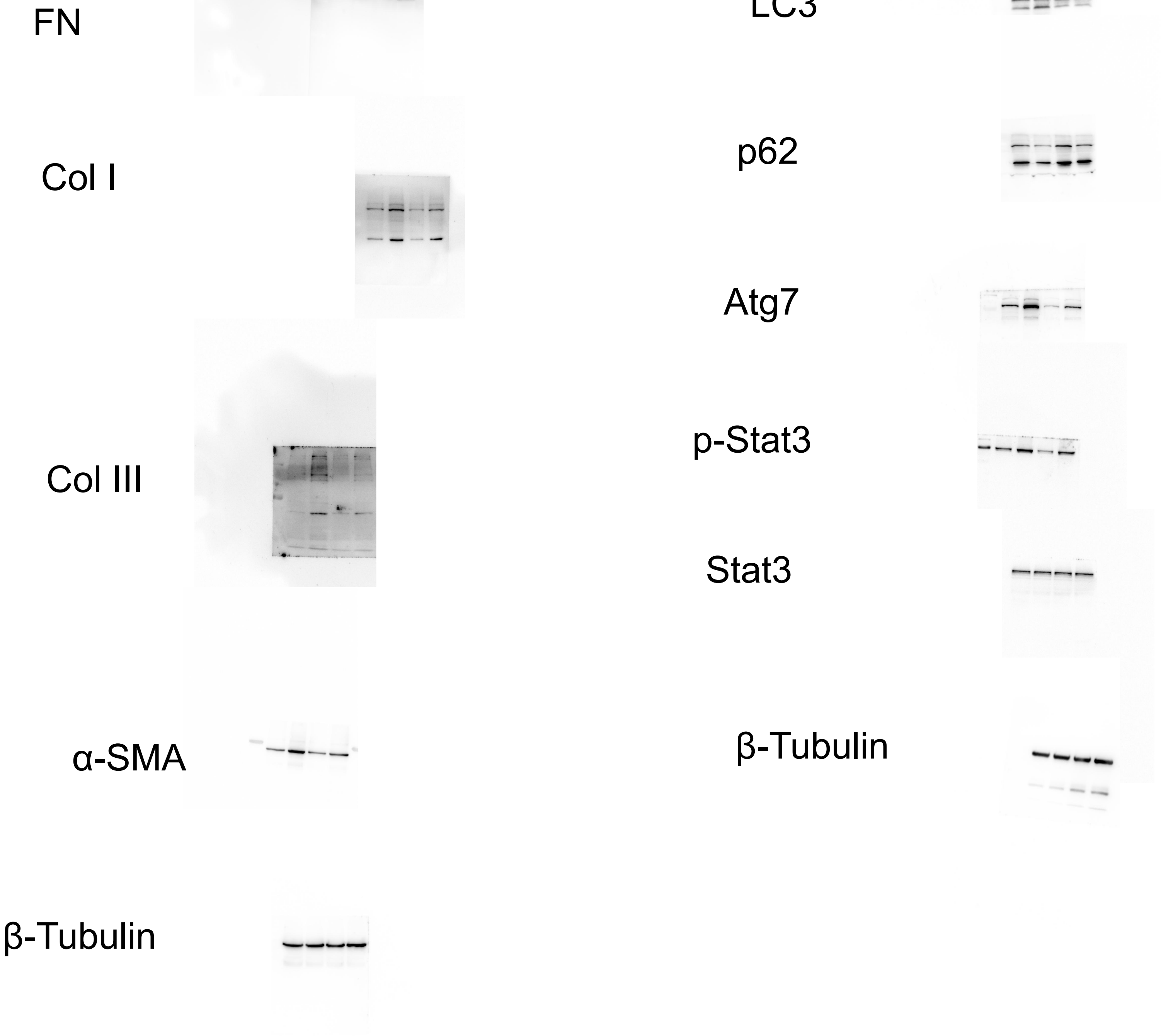
FN
LC3
Col I
p62
Atg7
p-Stat3
Col III
Stat3
α-SMA
β-Tubulin
β-Tubulin

Figure9

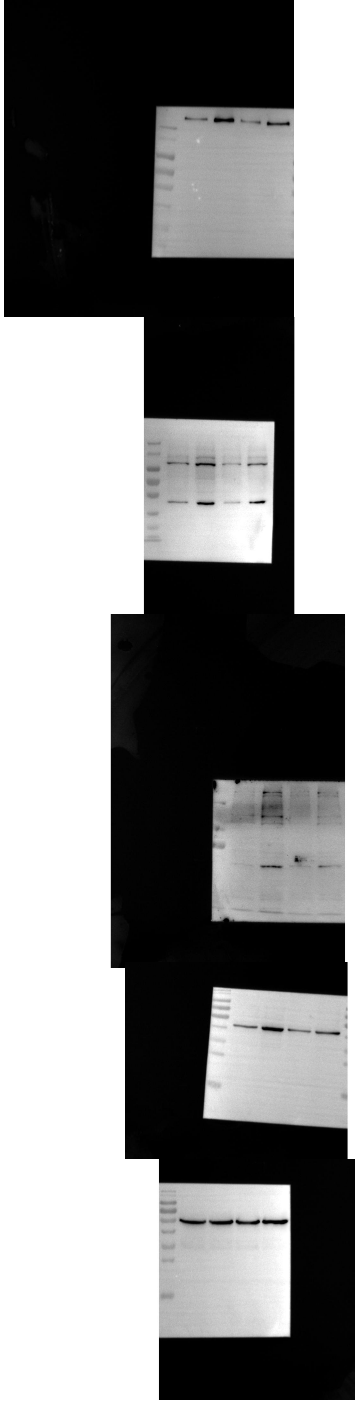

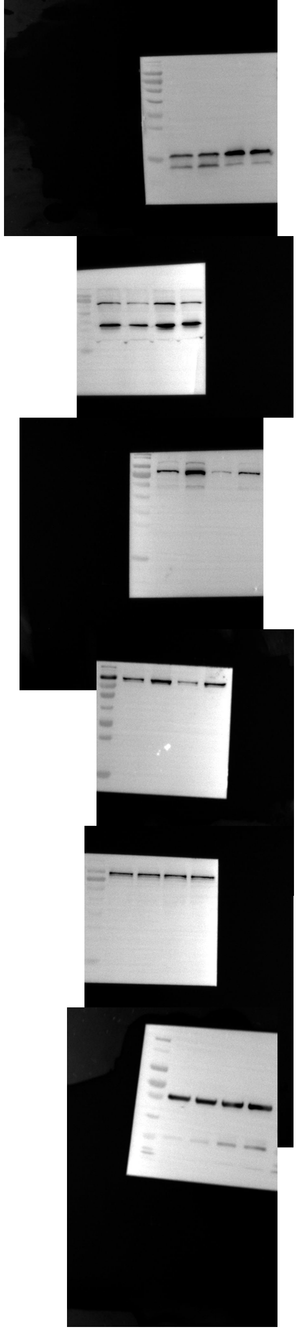

Supplementary Figure1

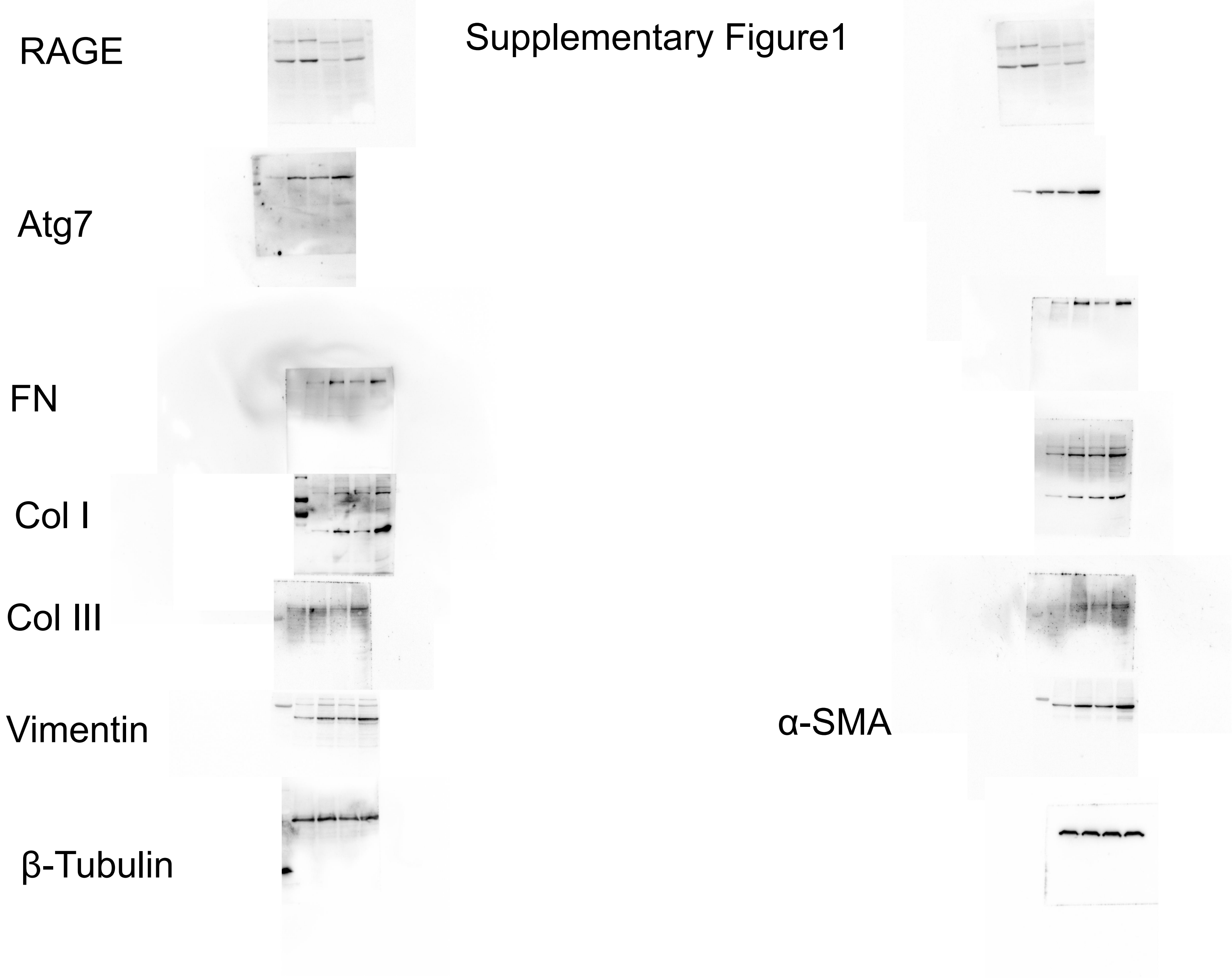

Supplementary Figure1

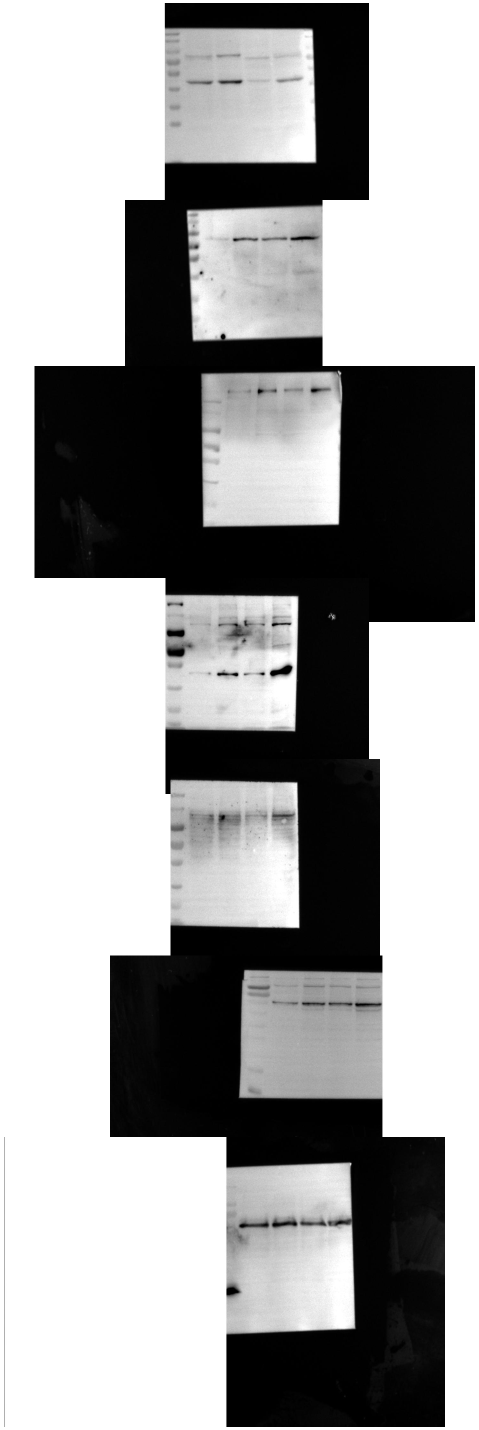

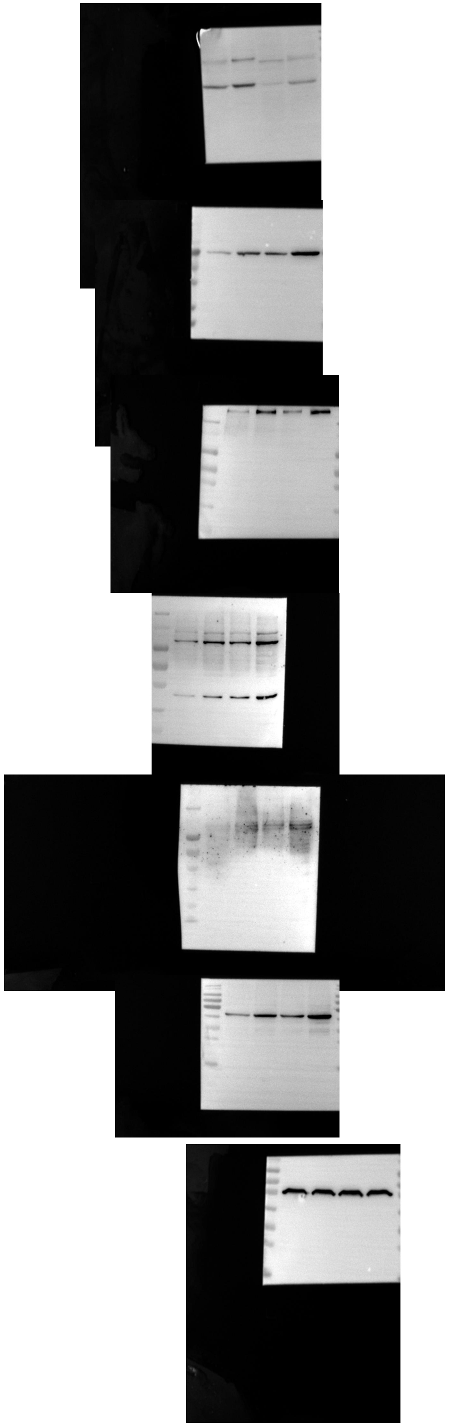

Supplementary Figure2

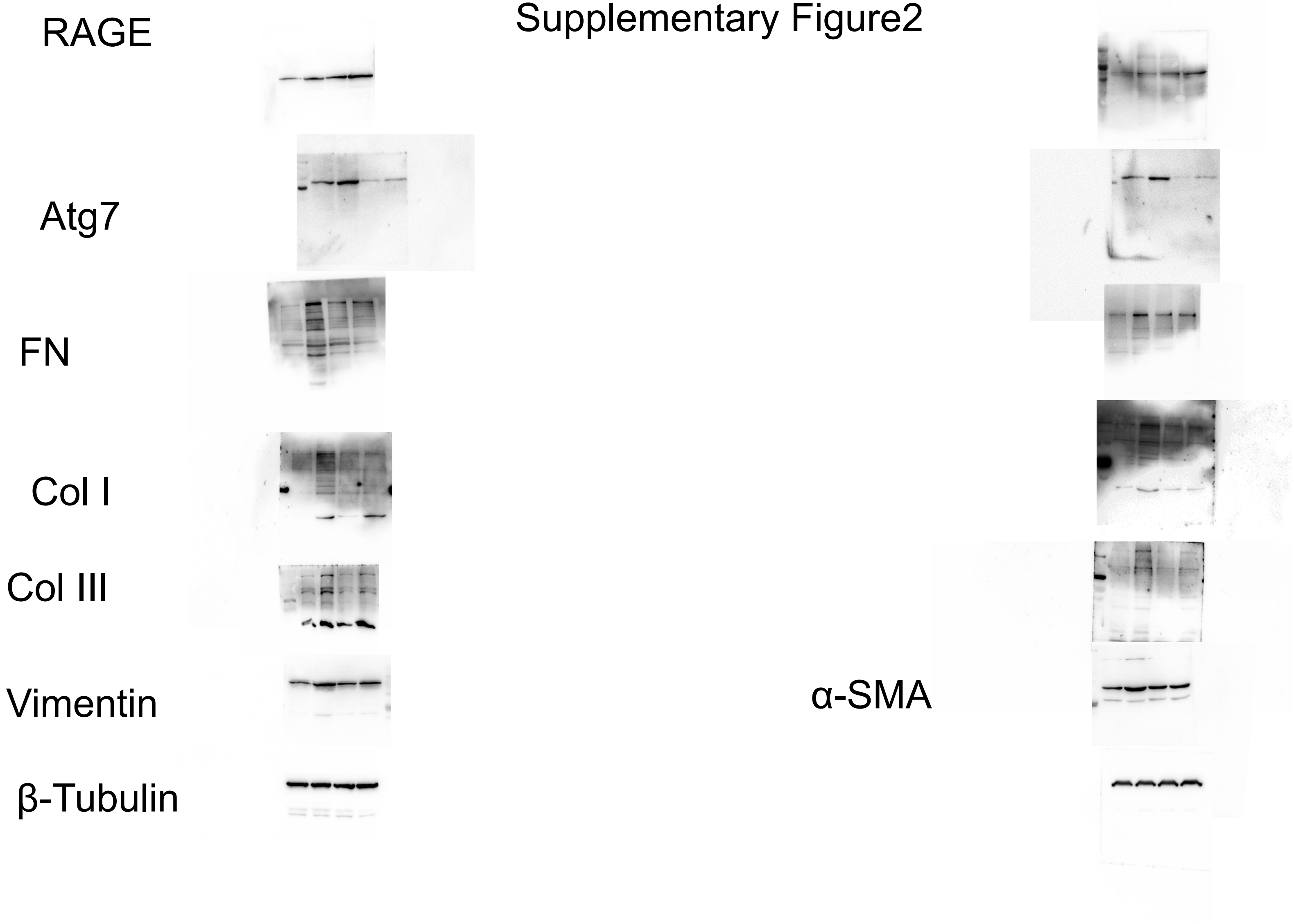

Supplementary Figure2

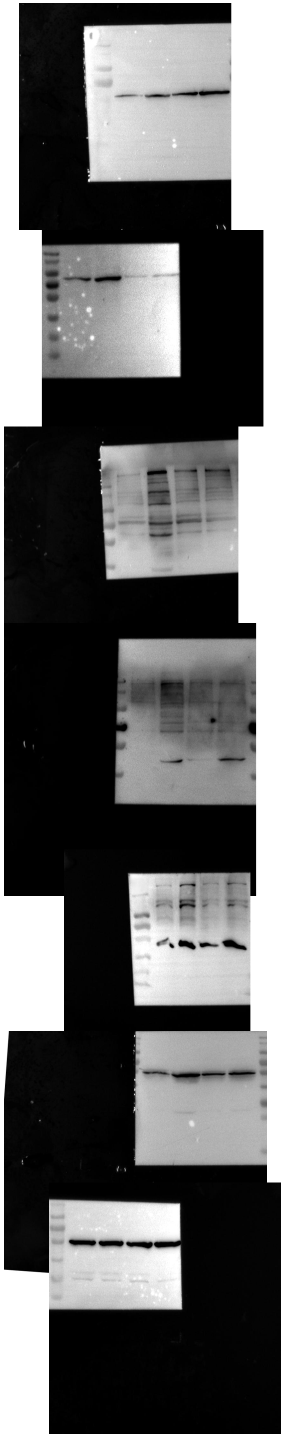

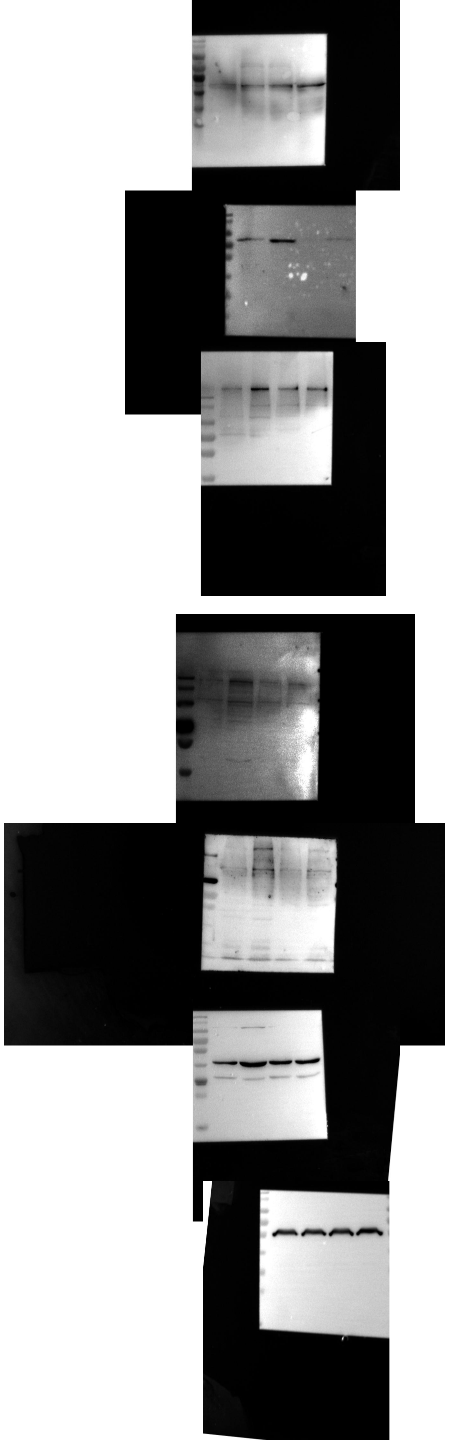

Supplementary Figure3

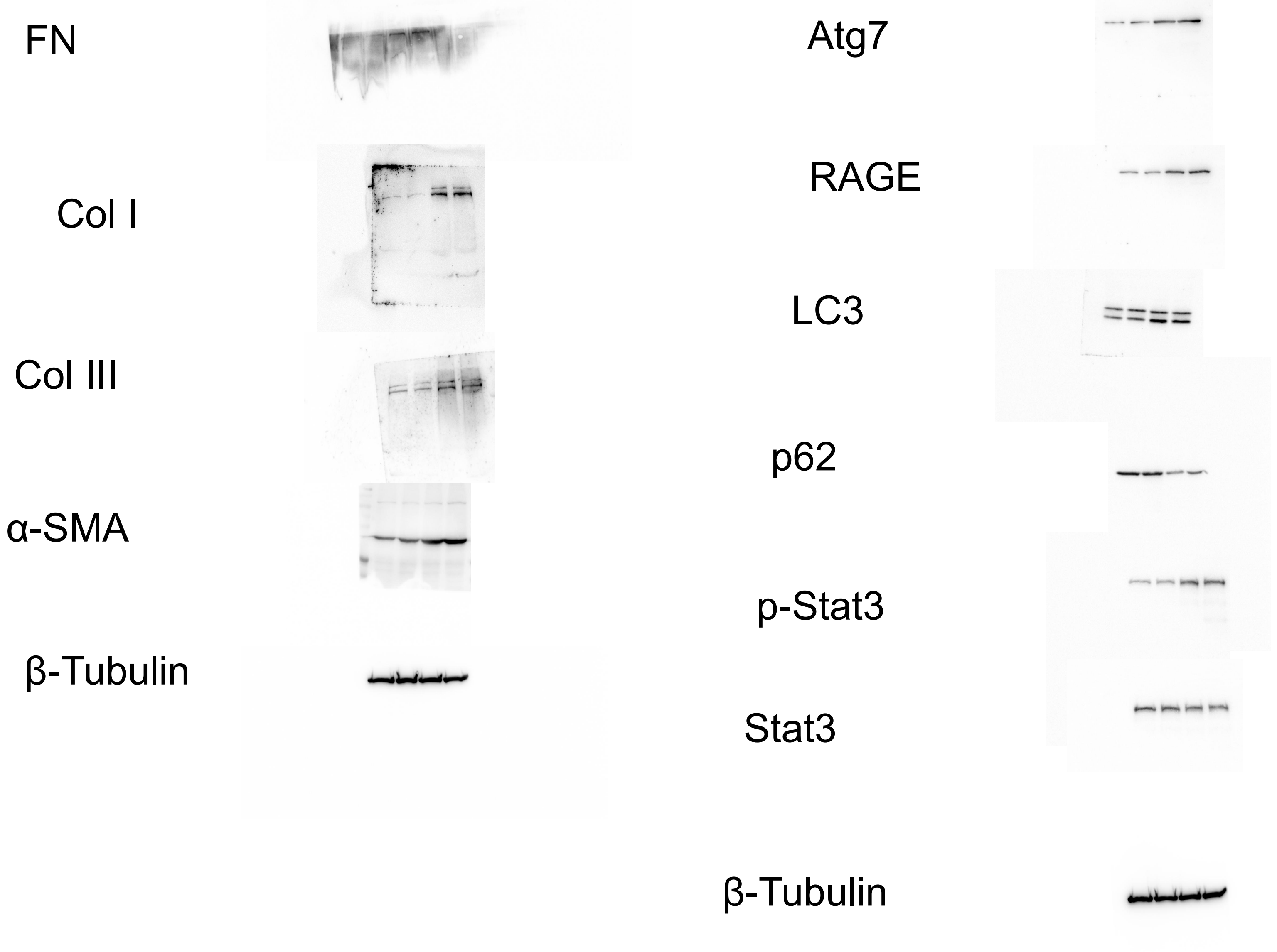

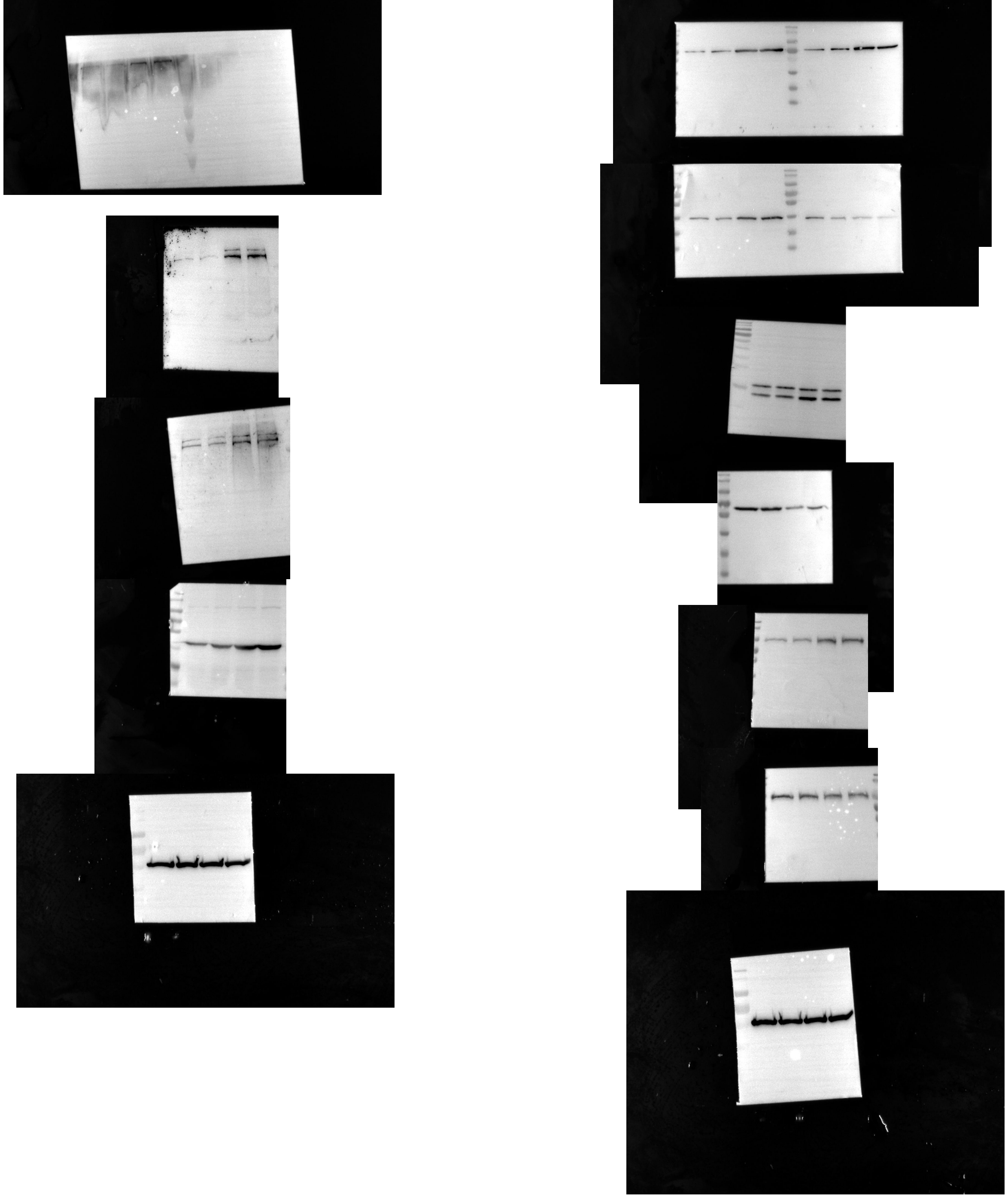

Supplementary Figure3
